# Metabolic Barriers to Cancer Immunotherapy: Multiscale Mechanisms from Organelle Dysfunction to Organ-Specific Constraints

**DOI:** 10.7150/ijbs.137618

**Published:** 2026-09-24

**Authors:** Zichen Gui, Xiaoyin Yuan, Xiaoping Chen, Qibo Huang, Bixiang Zhang, Junnan Liang

**Affiliations:** 1 Division of Hepato-Pancreato-Biliary Surgery, Tongji Hospital, Tongji Medical College, Huazhong University of Science & Technology, Wuhan, Hubei 430030, China.; 2 Key Laboratory of Organ Transplantation, Ministry of Education; NHC Key Laboratory of Organ Transplantation; Key Laboratory of Organ Transplantation, Chinese Academy of Medical Sciences, Wuhan, China.; 3 Hubei Key Laboratory of Hepato-Pancreato-Biliary Diseases, Wuhan, Hubei 430030, China.; 4 Clinical Medicine Research Centre for Hepatic Surgery of Hubei Province, Hubei 430030, China.; 5 Clinical Medicine Research Centre for Pancreatic Surgery of Hubei Province, Hubei 430030, China.; 6 Department of Breast and Thyroid Surgery, Union Hospital, Tongji Medical College, Huazhong University of Science and Technology, Wuhan, China.

**Keywords:** immunometabolism, immune checkpoint blockade, T cell exhaustion, tumor microenvironment, metabolic homeostasis

## Abstract

Immune checkpoint blockade (ICB) has reshaped cancer therapy, yet primary resistance and acquired relapse remain common despite therapeutic targeting of inhibitory receptor pathways. This limitation suggests that checkpoint release may remain insufficient when antitumor lymphocytes lack the metabolic, organelle-level, spatial, or systemic conditions required for tissue entry, functional recovery, and durable effector activity. In this review, we organize metabolic barriers to ICB into a multiscale framework comprising four interconnected axes. The Local Resource Axis captures how nutrient competition, enzymatic depletion, lipid dysregulation, suppressive metabolites, and ionic stress reshape the tumor microenvironment. The Organelle Axis examines mitochondrial dysfunction, endoplasmic reticulum stress, and intercellular mitochondrial transfer as determinants of T-cell functional recovery. The Spatial Axis addresses stromal, vascular, and organ-specific immune-metabolic niches that regulate immune access and site-specific resistance. The Systemic Axis integrates host metabolic and temporal states, including obesity, cachexia, microbial ecology, and circadian organization. Across these axes, metabolic constraints impair T-cell receptor (TCR) signaling, biosynthesis, redox homeostasis, organelle fitness, tissue infiltration, and host immune reserve, while also reducing malignant-cell susceptibility to immune-mediated killing. We further discuss therapeutic implications through a clinical-readiness and chrono-metabolic scheduling framework that distinguishes practice-ready vascular-targeted combinations from trial-prioritized or investigational dietary, microbiome-directed, metabolite-targeted, and engineered T-cell strategies. This evidence-calibrated perspective reframes tumor immunometabolism not as a static catalogue of targets, but as a set of biologically timed and compartment-specific constraints that determine the conditions under which ICB can achieve durable immune control.

## Introduction

The clinical success of immune checkpoint blockade (ICB) has established immunotherapy as a major pillar of cancer treatment. Antibodies targeting cytotoxic T-lymphocyte-associated protein 4 (CTLA-4), programmed cell death protein 1 (PD-1), or programmed death ligand 1 (PD-L1) can produce durable tumor control in selected patients, yet primary resistance and acquired relapse remain frequent across tumor types 1-10. These outcomes cannot be explained by the pharmacological relief of inhibitory receptor signaling alone. Checkpoint blockade can release suppressive signals, but it cannot by itself ensure that tumor-reactive T cells retain the metabolic reserve, organelle competence, tissue access, and systemic support required for expansion, tumor infiltration, cytotoxic activity, and persistence.

Tumor immunometabolism has therefore become central to understanding the limits of ICB. Within tumors, malignant, stromal, endothelial, myeloid, and lymphoid cells compete for nutrients and exchange metabolites across spatially heterogeneous niches. Glucose, amino acids, lipids, adenosine, lactate, and ions do not act merely as passive substrates or waste products. Instead, they can regulate T-cell receptor (TCR) signaling, cytokine translation, redox homeostasis, chromatin state, mitochondrial fitness, and susceptibility to ferroptosis 11-16. The therapeutic interpretation of these pathways is complicated by their compartment- and timing-dependent effects. The same metabolic pathway may support tumor growth, sustain effector T-cell function, enable regulatory-cell adaptation, or preserve host tissue homeostasis. Metabolic intervention in cancer immunotherapy therefore cannot be reduced to a simple strategy of inhibiting tumor metabolism or supplementing immune cells.

Several influential reviews have synthesized the rapidly expanding field of tumor immunometabolism 17-19. These pathway-centered discussions have clarified many key mechanisms, but they are less suited to explaining how metabolic resistance is organized across biological scales. A local nutrient deficit may impair intracellular organelle fitness. Organelle dysfunction may limit T-cell recovery after PD-1 blockade. Stromal and vascular architecture may determine whether metabolically competent lymphocytes can enter tumors. Host-level states such as obesity, cachexia, microbial ecology, and circadian timing may set the baseline conditions under which antitumor immunity operates. These relationships suggest that metabolic resistance to ICB should be understood not as a collection of isolated lesions, but as a multiscale architecture of interdependent constraints spanning local metabolic stress, organelle dysfunction, tissue-level exclusion, and systemic immune vulnerability.

In this review, we organize metabolic barriers to immunotherapy through four interconnected axes, as outlined in Figure 1. This framework emphasizes that ICB releases inhibitory receptor signaling but does not by itself ensure the metabolic, organelle-level, spatial, and systemic conditions required for durable antitumor immunity. The Local Resource Axis describes nutrient competition, enzymatic depletion, lipid dysregulation, suppressive metabolites, and ionic stress within the tumor microenvironment. The Organelle Axis examines mitochondrial dysfunction, endoplasmic reticulum stress, and intercellular mitochondrial transfer as determinants of T-cell metabolic reserve, functional recovery, and tumor-immune metabolic redistribution. The Spatial Axis addresses stromal, vascular, and organ-specific immune-metabolic niches that regulate lymphocyte entry, persistence, and site-specific resistance. The Systemic Axis considers how host metabolic and temporal states, including obesity, cachexia, gut microbial ecology, and circadian organization, reshape immune-cell reserve and checkpoint responsiveness. Table 1 summarizes the dominant resistance modules, primary cellular or tissue compartments, mechanistic bridges to ICB failure, evidence boundaries, and translational implications across these four axes. We then translate these mechanisms into a therapeutic prioritization framework that integrates clinical readiness, patient safety, biological matching, and chrono-metabolic scheduling. By linking metabolic lesions to their dominant biological compartment, evidentiary maturity, and timing relative to ICB administration or adoptive T-cell manufacturing, this review aims to move tumor immunometabolism from a descriptive catalogue of resistance mechanisms toward a framework for biologically timed and evidence-calibrated therapeutic design.

## Local resource axis: nutrient competition, metabolite signaling, and chemical stress

The Local Resource Axis describes how extracellular nutrient availability, metabolite accumulation, lipid exposure, and ionic imbalance shape the metabolic conditions under which antitumor immunity operates. In the tumor microenvironment (TME), these pressures do not act as a single form of “nutrient deprivation.” Instead, they include context-dependent competition for glucose and glutamine, active enzymatic depletion of selected amino acids, lipid-specific toxicity and ferroptotic vulnerability, and the accumulation of bioactive metabolites or ions that directly alter immune signaling. These constraints are particularly relevant to ICB, because reinvigorated T cells must not only receive permissive receptor signals but also obtain substrates, maintain redox balance, preserve membrane and chromatin integrity, and sustain effector function within metabolically hostile tumor niches. This section therefore organizes local metabolic resistance into four interrelated categories: glucose dysregulation, amino acid dysregulation, lipid dysregulation, and microenvironmental chemical constraints.

## Glucose dysregulation: nutrient partitioning, glycolysis-dependent immune resistance, and lactate adaptation

Glucose dysregulation constrains antitumor immunity through three distinct but interacting processes: heterogeneous nutrient partitioning, glycolysis-dependent regulation of T-cell and tumor-cell function, and cell-state-specific adaptation to extracellular lactate. Following TCR stimulation, activated effector T cells rapidly increase aerobic glycolysis alongside mitochondrial metabolism to support biosynthesis, clonal expansion, and acute cytokine production 14, 20. In mouse sarcoma models, highly glycolytic tumor cells reduced extracellular glucose availability and suppressed T cell mechanistic target of rapamycin complex 1 (mTORC1) activity, glycolytic capacity, and interferon-gamma (IFN-γ) production 13. However, cell-resolved tracer studies across several mouse tumor models showed that myeloid cells had the greatest cell-intrinsic capacity for glucose uptake, whereas cancer cells preferentially acquired glutamine 12. Glucose restriction should therefore be viewed as a context-dependent constraint shaped by tumor type, cellular composition, and spatial nutrient partitioning, rather than as a universal consequence of tumor glycolysis.

Within T cells, glycolytic flux supports effector function through both metabolic substrate supply and direct regulation of signaling. The glycolytic intermediate phosphoenolpyruvate sustains calcium-dependent nuclear factor of activated T cells (NFAT) signaling, thereby linking glucose metabolism to TCR signal transduction 11. When glyceraldehyde-3-phosphate dehydrogenase (GAPDH) is not engaged in glycolysis, it binds the 3′ untranslated region of IFN-γ messenger RNA and suppresses its translation 15. These mechanisms establish glucose metabolism as a regulator of TCR signaling and cytokine translation in addition to its bioenergetic and biosynthetic functions.

Tumor-cell glycolysis can independently promote immune resistance by reducing malignant-cell susceptibility to immune-mediated killing. Genome-wide clustered regularly interspaced short palindromic repeats (CRISPR) screening showed that loss of glucose transporter 1 (GLUT1, encoded by SLC2A1) or glucose-6-phosphate isomerase (GPI1) increased tumor-cell killing by cytotoxic T cells. Mechanistically, GLUT1 inactivation redirected tumor-cell metabolism toward oxidative phosphorylation, increased reactive oxygen species, and sensitized malignant cells to tumor necrosis factor alpha (TNF-α)-mediated bystander killing. Genetic or pharmacological GLUT1 inhibition also enhanced antitumor immunity and improved the efficacy of PD-1 blockade in the corresponding mouse models 21. Tumor glycolysis therefore affects immune control not only by altering nutrient availability, but also by determining how readily malignant cells succumb to immune effector mechanisms.

As a downstream product of glycolysis, extracellular lactate produces additional immune effects that depend on cell differentiation state and transporter expression. In mouse melanoma models, lactate dehydrogenase A-dependent lactic acid production reduced T cell and natural killer-cell infiltration and IFN-γ production 22. Regulatory T cells adapted to glucose-poor, lactate-rich tumors by importing lactate through monocarboxylate transporter 1 (MCT1), promoting NFAT1-dependent PD-1 expression and reducing the efficacy of PD-1 blockade in highly glycolytic tumors 23. Terminally exhausted CD8^+^ T cells exhibited a distinct response through selective upregulation of monocarboxylate transporter 11 (MCT11), encoded by SLC16A11. Conditional MCT11 deletion or antibody blockade reduced lactate uptake and metabolism, improved exhausted T cell polyfunctionality, and inhibited tumor growth. In the MC38 model, combining MCT11 blockade with PD-1 blockade increased the frequency of complete tumor responses compared with PD-1 blockade alone 24. Lactate exposure therefore does not generate a uniform immune phenotype. Instead, its consequences depend on immune-cell identity, differentiation state, transporter expression, and metabolic capacity.

Together, these findings show that glucose dysregulation acts through heterogeneous nutrient allocation, direct glycolytic control of both T-cell signaling and tumor-cell killing sensitivity, and transporter-dependent responses to extracellular lactate. In selected preclinical models, these mechanisms constrained responses to immune checkpoint blockade, but their relative importance is likely to vary across tumor types. This section treats lactate primarily as an extracellular metabolite and transport substrate, whereas its translation into longer-lasting chromatin programs through histone lactylation is considered separately below. Whereas glucose dysregulation primarily alters acute metabolic flux, signaling, and immune-cell adaptation, amino acid dysregulation extends metabolic suppression through active enzymatic depletion, bioactive metabolite signaling, and methylation-dependent regulation.

## Amino acid dysregulation: nutrient competition, metabolite signaling, and epigenetic reprogramming

Amino acid dysregulation can constrain antitumor immunity and durable responses to ICB through several mechanistically distinct processes rather than a uniform deprivation program. These include competition for biosynthetic substrates, enzymatic nutrient depletion, the generation of bioactive metabolites, and disruption of methyl-donor-dependent regulation. Glutamine limitation mainly restricts anabolic metabolism and nutrient-responsive signaling, whereas arginine depletion weakens TCR signaling and metabolic fitness. Tryptophan catabolism couples amino acid stress to kynurenine-mediated receptor signaling, whereas methionine dysregulation combines methyl-donor limitation with suppression by accumulated methionine-cycle metabolites. Because these lesions differ in their cellular sources, mechanistic basis, and reversibility, amino-acid-directed interventions cannot be reduced to a general strategy of supplementation or restriction.

### Glutamine limitation: disrupted biosynthesis and nutrient sensing

Glutamine limitation can impair antitumor T-cell responses by restricting both biosynthetic metabolism and amino acid-responsive signaling, although the extent of this constraint varies across tumor types. Following TCR activation, T cells increase glutamine uptake and use glutamine-derived carbon and nitrogen to support tricarboxylic acid cycle anaplerosis, nucleotide and amino acid synthesis, polyamine production, and redox balance 14, 25, 26. In MYC-driven cancer models, oncogenic MYC can induce glutaminolysis and cellular dependence on glutamine 27. Independent work further showed that tumor cells and cDC1s compete for glutamine through SLC38A2, with tumor-cell SLC38A2 loss increasing intratumoral glutamine availability and strengthening antitumor immunity 28. Consistent with a competition model in glutamine-dependent triple-negative breast cancer, tumor-cell-specific glutaminase (GLS) deletion increased interstitial glutamine availability and enhanced T-cell infiltration and effector activity 29. These findings support competition for glutamine in selected tumors but do not establish severe glutamine depletion as a universal property of the tumor microenvironment.

The vulnerability of activated T cells to reduced glutamine availability reflects their dependence on glutamine for both biosynthetic metabolism and nutrient-responsive signaling. Glutamine withdrawal directly limits T-cell proliferation and cytokine production in culture 25. Mechanistically, TCR signaling induces MYC-dependent programs that coordinate glutamine catabolism and polyamine biosynthesis required for cell growth and proliferation 14. In primary CD4^+^ T cells, SLC1A5-mediated glutamine uptake supported TCR-induced mTORC1 activation and inflammatory T-cell differentiation 30. Separately, SLC7A5-mediated uptake of leucine and other large neutral amino acids was required to sustain mTORC1 activity, c-MYC protein expression, and metabolic reprogramming in activated T cells 31. A bidirectional model in which SLC1A5-dependent glutamine uptake supports glutamine efflux through SLC7A5/SLC3A2 in exchange for leucine influx was demonstrated in non-lymphoid cell lines 32, but it has not been established as the obligatory link between tumor-cell glutamine consumption and T-cell dysfunction *in vivo*. Reduced glutamine availability may therefore impair T-cell function through combined effects on substrate supply, amino acid transport, and mTORC1-MYC-dependent metabolic reprogramming, although the relative contribution of these pathways within tumors remains unresolved. Whereas glutamine limitation depends strongly on tumor-cell nutrient demand, arginine depletion can be actively imposed by catabolic enzymes expressed by tumor-associated myeloid cells.

### Arginine depletion: impaired T-cell receptor signaling and metabolic fitness

Extracellular L-arginine can be actively depleted by arginase 1 (ARG1)-expressing tumor-associated myeloid cells. T cells are particularly susceptible because their basal capacity for endogenous arginine synthesis is limited by low expression of argininosuccinate synthase 1 (ASS1) and ornithine transcarbamylase (OTC) 33. In the 3LL Lewis lung carcinoma model, tumor-derived mature myeloid cells coexpressing ARG1 and cationic amino acid transporter 2B (CAT-2B) rapidly imported and depleted extracellular L-arginine *in vitro* 34. Complementary macrophage cultures showed that interleukin-4 (IL-4) and interleukin-13 (IL-13) induced ARG1 and CAT-2B and reduced extracellular arginine 35. In patients with metastatic renal cell carcinoma, elevated arginase activity was concentrated in CD11b^+^CD14^-^CD15^+^ granulocytic myeloid-derived suppressor cells and was associated with lower plasma L-arginine 36. These findings support myeloid ARG1 as an active mechanism of arginine depletion, while indicating that the dominant ARG1-expressing population and the site of depletion vary across tumor contexts.

Reduced arginine availability can impair TCR signaling, but the available evidence does not establish a single uninterrupted ARG1-CD3ζ pathway across all models. In macrophage-T cell cultures and in T cell or Jurkat-cell systems, macrophage-mediated arginine consumption or direct arginine withdrawal reduced expression of the CD3 zeta chain (CD3ζ, encoded by CD247) 35, 37, 38. In the 3LL model, ARG1-expressing tumor-derived myeloid cells depleted arginine, prevented CD3ζ re-expression after T cell stimulation, and inhibited antigen-specific OT-I and OT-II T-cell proliferation 34. Patient evidence remained associative: elevated arginase activity, reduced plasma arginine, and low CD3ζ expression were observed together in metastatic renal cell carcinoma 36. The role of CD3ζ in coupling the TCR to downstream signal transduction was established independently in a structure-function study 39. Systemic or myeloid-specific Arg1 deletion in 3LL tumor-bearing mice improved antigen-driven T-cell proliferation and inhibited tumor growth 40, providing *in vivo* evidence for ARG1-dependent immunosuppression without establishing CD3ζ loss as its sole mediator. Tumor-derived macrophage oxidative stress can also suppress CD3ζ independently 41, indicating that several tumor-microenvironmental stresses may converge on this signaling defect.

Arginine availability also regulates T cell metabolic fitness and adaptive capacity. Activated human T cells can induce ASS1 and import citrulline through L-type amino acid transporter 1 (LAT1, encoded by SLC7A5). thereby regenerating arginine and restoring proliferation under arginine-limited culture conditions 42. Separately, increasing intracellular arginine shifted activated T cell metabolism from glycolysis toward oxidative phosphorylation (OXPHOS), enhanced survival, and favored a central memory-like phenotype with improved antitumor activity 43. These studies show that dependence on extracellular arginine is conditional and therapeutically modifiable, but they should not be interpreted as direct reversal experiments for the ARG1-CD3ζ mechanism. Overall, ARG1-mediated depletion can constrain both TCR signaling and metabolic fitness, whereas the magnitude and reversibility of these effects depend on the myeloid source of arginase, local citrulline availability, and the metabolic state of responding T cells. Unlike arginine depletion, tryptophan catabolism combines substrate loss with the generation of immunoregulatory metabolites.

### Tryptophan catabolism: nutrient stress and metabolite signaling

Tryptophan catabolism differs from simple nutrient competition because it combines reduced substrate availability with the generation of bioactive kynurenine-pathway metabolites. Indoleamine 2,3-dioxygenase 1 (IDO1) expression by tumor cells can confer resistance to immune-mediated tumor rejection, while a distinct subset of IDO1-expressing human dendritic cells suppresses T-cell proliferation 44, 45. Tryptophan 2,3-dioxygenase (TDO2) provides a separate tumor-associated route into the kynurenine pathway, and its pharmacological inhibition reversed tumor immune resistance in preclinical models 46. Thus, although IDO1 and TDO2 differ in their cellular sources and regulatory contexts, both can reduce tryptophan availability while increasing the production of kynurenine-pathway metabolites.

Amino acid stress sensing represents one potential consequence of this metabolic shift. In systems involving IDO1-expressing plasmacytoid dendritic cells, IDO1 activity activated general control nonderepressible 2 (GCN2) signaling in responding T cells, and Gcn2 deficiency prevented proliferative arrest and anergy 47. However, T-cell-specific Gcn2 deletion did not improve tumor control or alter tumor-infiltrating T-cell responses in the B16 melanoma model. Even after enforced Tdo expression, intratumoral tryptophan remained above the concentration required for GCN2 activation 48. These findings indicate that GCN2 can mediate T-cell suppression when tryptophan restriction is sufficiently severe, but it is not a universal consequence of tumor-associated tryptophan catabolism.

Nutrient stress can also enhance the effects of kynurenine-pathway metabolites. Tryptophan depletion increased aryl hydrocarbon receptor (AHR) expression independently of GCN2, while GCN2-dependent induction of LAT1 increased kynurenine uptake. Together, these changes enhanced AHR activation and regulatory T (Treg) cell differentiation *in vitro* 49. Kynurenine can activate the human AHR in tumor-associated settings 50, and murine T cell studies further demonstrated AHR-dependent generation of forkhead box P3 (FOXP3)^+^ Treg cells 51. In IDO1- or TDO2-overexpressing tumor models, AHR signaling supported a suppressive interaction between Treg cells and tumor-associated macrophages and impaired CD8^+^ T cell effector function. Selective AHR inhibition delayed tumor progression and enhanced the antitumor effects of PD-1 blockade in these preclinical models 52. Overall, tryptophan catabolism can suppress antitumor immunity through both nutrient stress and kynurenine-AHR signaling, with their relative contribution determined by the degree of tryptophan depletion, the dominant catabolic enzyme, and the cellular context 53. Whereas tryptophan catabolism combines amino acid stress with metabolite-receptor signaling, methionine dysregulation additionally alters methyl-donor-dependent regulation and exposes T cells to methionine-cycle metabolites.

### Methionine dysregulation: methyl-donor depletion and metabolite-mediated immunosuppression

Within the tumor microenvironment, methionine dysregulation can suppress antitumor immunity through two mechanistically distinct processes: competition that limits T cell methionine and methyl-donor availability, and accumulation of tumor-derived methionine-cycle metabolites. Activated T cells are particularly vulnerable because TCR engagement increases methionine transport and methionine-cycle flux, sustaining S-adenosylmethionine (SAM)-dependent RNA, protein, and histone methylation 54. In coculture and mouse models, tumor-cell SLC43A2 lowered methionine and SAM in CD8^+^ T cells, reducing histone H3 lysine 79 dimethylation (H3K79me2), signal transducer and activator of transcription 5 (STAT5) expression and signaling, survival, and effector function. Human colorectal cancer samples showed concordant associations 55. Related work in CD4^+^ T cells linked cancer-cell methionine consumption to reduced H3K79me2 and AMP-activated protein kinase expression, increased endoplasmic reticulum stress, and progressive PD-1 upregulation 56. Preclinical studies further suggested that methionine availability during initial TCR engagement influences later CD8^+^ T cell exhaustion through arginine methylation of KCa3.1 and calcium-NFAT1 signaling 57. Thus, both the extent and timing of methionine deprivation may shape T cell fate through distinct methylation-dependent mechanisms.

Methionine dysregulation is not limited to nutrient scarcity. In hepatocellular carcinoma, patient multi-omics analyses and experimental models linked reprogrammed tumor methionine recycling to increased SAM and 5-methylthioadenosine (MTA), T cellexhaustion, and impaired immune control. Tumor-cell MAT2A deletion reduced T-cell dysfunction and tumor growth in mice 58. Clinical analyses linked 9p21/MTAP loss to reduced tumor-infiltrating lymphocytes and resistance to ICB, whereas mechanistic models implicated extracellular MTA accumulation. Enzymatic MTA depletion restored T cell activity and improved checkpoint blockade in MTAP-deficient preclinical models 59. These findings extend the mechanism from competition for a limiting nutrient to suppression by accumulated metabolites, although they do not establish that both processes coexist or contribute equally within the same tumor.

This distinction is essential for interpreting intervention studies. In colorectal cancer models, dietary methionine restriction enhanced PD-1 blockade by reducing tumor-cell messenger RNA N6-methyladenosine (m6A) modification and YTHDF1-dependent translation of PD-L1 and V-domain immunoglobulin suppressor of T-cell activation (VISTA) 60. By contrast, systemic methionine restriction reduced T cell abundance and impaired antitumor immunity and immunotherapy in immunocompetent intestinal and colorectal tumor models through sulfur deficiency and microbiota-dependent loss of hydrogen sulfide 61. Methionine supplementation during early T-cell activation also reduced T cell exhaustion and complemented PD-1 blockade in selected mouse models 57.

These divergent outcomes cannot be reduced to a simple restriction-versus-supplementation rule. Rather, they appear to depend on which biological response predominates, when the intervention is applied, the tumor genotype, and host-microbiota metabolism. Methionine metabolism should therefore be viewed as a timing- and context-dependent regulator of the tumor immune microenvironment, rather than a uniformly actionable nutrient axis.

Together, these findings show that amino-acid-directed interventions cannot be reduced to simple supplementation or restriction. Their immunological effects depend on the dominant metabolic lesion, the responsible cell population, and the timing of intervention. Accordingly, they may either enhance or undermine the efficacy of immune checkpoint blockade. This context dependence distinguishes amino acid dysregulation from the lipid axis, in which lipid identity, cellular accumulation, and oxidative handling determine whether T cells adapt metabolically or progress toward lipotoxicity and ferroptotic death.

## Lipid dysregulation: metabolic adaptation, lipotoxicity, and ferroptotic vulnerability

Unlike the predominantly deprivation-based constraints imposed by glucose and amino acids, lipid dysregulation in the TME represents a heterogeneous challenge rather than uniform substrate excess. The consequences of intratumoral lipid exposure depend on lipid species and oxidation state, their cellular source and distribution, and the capacity of tumor-infiltrating CD8^+^ T cells to acquire, traffic, oxidize, and buffer the available substrates. Fatty acids can therefore support metabolic adaptation under glucose-poor and hypoxic conditions, whereas selected saturated or oxidized lipids can disrupt mitochondrial function, reinforce exhaustion, and increase lipid-peroxidation stress. When lipid and iron loading exceed glutathione-dependent antioxidant defenses and phospholipid homeostasis, this stress can progress to ferroptotic cell death. These context-dependent responses reduce the metabolic fitness and functional persistence of antitumor T cells and may thereby limit the depth and durability of immune checkpoint blockade.

### Intratumoral lipid exposure: lipid-specific toxicity and T cell metabolic adaptation

At the level of individual lipid species, the functional outcome of intratumoral lipid exposure depends on both substrate identity and the capacity of CD8^+^ T cells to acquire and oxidize the available fatty acids.

Specific long-chain fatty acids, particularly palmitate, can suppress CD8^+^ T cells through signaling-dependent exhaustion or accumulation-associated lipotoxicity. In Riplet-deficient hepatocellular carcinoma (HCC), promoter hypermethylation-associated Riplet silencing reduced K48-linked polyubiquitination of fatty acid synthase (FASN), increasing tumor-cell fatty-acid production. Tumor-derived palmitic acid enhanced signal transducer and activator of transcription 3 (STAT3) palmitoylation in CD8+ T cells, promoted terminal exhaustion, and contributed to resistance to PD-1 blockade. FASN inhibition improved treatment responsiveness in the corresponding models 62. In pancreatic ductal adenocarcinoma (PDAC), long- and very-long-chain fatty-acid-containing lipids accumulated in CD8^+^ T cell-rich tumor regions, while specific long-chain fatty acids progressively accumulated within these cells. Palmitate impaired mitochondrial function and effector activity, whereas reduced expression of very-long-chain acyl-CoA dehydrogenase (VLCAD, encoded by ACADVL) limited fatty-acid oxidation and amplified lipotoxicity. Enforced ACADVL expression improved the survival and persistence of engineered tumor-specific T cells 63. Together, these studies show that palmitate can impair CD8^+^ T cells through distinct mechanisms, although only the Riplet-deficient hepatocellular carcinoma study directly established resistance to PD-1 blockade.

Beyond lipid identity, T cell outcome also depends on whether available fatty acids can be efficiently accessed and oxidized. In ovarian cancer models, tumor-induced endoplasmic reticulum stress activated spliced X-box-binding protein 1 (XBP1s) and suppressed transgelin 2 (TAGLN2), reducing the localization of fatty-acid-binding protein 5 (FABP5) to the CD8^+^ T-cell surface. This restricted fatty-acid uptake, mitochondrial respiration, and effector function, whereas TAGLN2-overexpressing chimeric antigen receptor (CAR) T cells showed improved therapeutic activity 64. By contrast, in hypoglycemic and hypoxic melanoma models, CD8^+^ tumor-infiltrating lymphocytes increased peroxisome proliferator-activated receptor alpha (PPARα)-dependent fatty-acid catabolism to partially preserve their function. Pharmacological enhancement of this adaptive program improved tumor control and synergized with PD-1 blockade 65. Thus, the immune consequences of intratumoral lipids are determined by the interaction among lipid composition, cellular accumulation and access, intracellular storage, and oxidative capacity rather than by abundance alone. This framework provides a transition to the mechanisms through which maladaptive lipid uptake and redox failure promote lipid peroxidation and ferroptotic vulnerability.

### Maladaptive lipid uptake and redox failure: ferroptotic vulnerability

Ferroptotic vulnerability in tumor-infiltrating CD8^+^ T cells does not arise from lipid abundance alone, but from the convergence of pro-oxidant lipid and iron loading with impaired antioxidant defense and phospholipid homeostasis. In mouse tumor models and human melanoma samples, dysfunctional CD8^+^ tumor-infiltrating lymphocytes upregulated cluster of differentiation 36 (CD36) and accumulated oxidized lipids. CD36-dependent uptake of oxidized low-density lipoprotein (OxLDL) increased lipid peroxidation and p38 mitogen-activated protein kinase signaling, whereas Cd36 deletion, glutathione peroxidase 4 overexpression, antioxidant treatment, or p38 inhibition preserved effector function in the corresponding experimental systems 66. These experiments established an OxLDL-CD36-p38 pathway of functional impairment but did not, by themselves, demonstrate ferroptotic cell death. In complementary mouse tumor models, CD36-mediated uptake of fatty acids, including arachidonic acid, promoted lipid peroxidation and ferroptosis; genetic Cd36 deletion or pharmacological ferroptosis inhibition restored cytokine production and improved the response to PD-1 blockade 67. Early-stage hepatocellular carcinoma models further showed that CD36 can connect oxidized-lipid sensing to iron loading. OxLDL-induced p38-CCAAT/enhancer-binding protein beta signaling increased transferrin receptor 1 expression and intracellular ferrous iron, whereas constitutive activation of nuclear factor erythroid 2-related factor 2 reduced lipid peroxidation and preserved CD8^+^ T-cell function 68. Thus, CD36 can increase ferroptosis-relevant oxidative pressure through both lipid uptake and iron accumulation, although the iron-regulatory mechanism has so far been established primarily in early-stage hepatocellular carcinoma.

Ferroptotic susceptibility is further amplified when glutathione synthesis and membrane-phospholipid homeostasis fail. In B16F10 melanoma models, tumor cells with high SLC7A11 expression outcompeted CD8^+^ T cells for cystine, thereby limiting T cell glutathione synthesis and increasing oxidative stress, CD36 expression, oxidized-lipid uptake, and ferroptosis. Tumor-cell Slc7a11 knockdown, intratumoral cystine supplementation, or enforced glutamate-cysteine ligase catalytic subunit expression in T cells reduced ferroptosis and improved T cell antitumor activity 69. A separate mechanism was identified in lung cancer, where intratumoral CD8^+^ T cells exhibited reduced phospholipid phosphatase 1 (PLPP1) expression and lower phosphatidylcholine and phosphatidylethanolamine levels. T-cell-specific Plpp1 loss increased susceptibility to unsaturated-fatty-acid-induced ferroptosis. Mechanistically, PD-1 signaling induced GATA-binding factor 1 (GATA1) binding to the Plpp1 promoter and suppressed Plpp1 expression. PD-1 blockade increased Plpp1 expression and restored antitumor function but did not rescue Plpp1-deficient T cells 70. Together, these studies identify three experimentally distinct but convergent routes that increase lipid peroxidation and ferroptotic vulnerability: CD36-dependent lipid and iron loading, cystine-limited glutathione synthesis, and PD-1-dependent disruption of phospholipid homeostasis. Their relative contributions are likely to vary across tumor types and T cell states.

### Fatty-acid desaturation and cholesterol homeostasis: tumor survival and T-cell dysfunction

Fatty-acid desaturation and cholesterol handling exert cell-specific effects: stearoyl-CoA desaturase 1 (SCD1)-derived monounsaturated fatty acids can protect tumor cells from ferroptosis, whereas SCD1 activity in CD8^+^ T cells can restrain effector function by promoting cholesterol esterification. In ovarian cancer models, SCD1 increased monounsaturated fatty acid synthesis and protected tumor cells from lipid peroxidation and ferroptotic death 71. In human and mouse CD8+ T cells, SCD1-derived oleic acid supported cholesterol esterification mediated by sterol O-acyltransferase 1 (SOAT1), commonly referred to in this literature as acyl-CoA:cholesterol acyltransferase 1 (ACAT1). Pharmacological SCD1 inhibition reduced oleic acid and esterified cholesterol and increased IFN-γ, tumor necrosis factor-alpha, and granzyme B production. Similar metabolic and functional changes were observed in tumor-infiltrating CD8^+^ T cells from MCA205-bearing mice 72.

The downstream consequence of cholesterol esterification is altered plasma-membrane organization and TCR signaling. Genetic or pharmacological inhibition of ACAT1 redirected cholesterol toward the plasma membrane, enhanced TCR clustering and immunological-synapse formation, and improved CD8^+^ T-cell proliferation and cytotoxicity. In mouse melanoma models, the ACAT inhibitor avasimibe also enhanced the antitumor activity of PD-1 blockade 73. These findings identify the distribution of cholesterol between esterified intracellular stores and the plasma membrane as an actionable determinant of T cell responsiveness.

Cholesterol can impair CD8^+^ T cells at either extreme of cellular availability. In selected tumor models, cholesterol accumulation activated endoplasmic reticulum stress and XBP1s signaling, increased inhibitory-receptor expression, and promoted CD8^+^ T cell exhaustion 74. By contrast, a cholesterol atlas of the tumor microenvironment showed that intratumoral T cells can become cholesterol deficient despite cholesterol enrichment in tumor cells and immunosuppressive myeloid populations. Tumor-associated oxysterols disrupted liver X receptor (LXR) and sterol regulatory element-binding protein 2 (SREBP2) signaling, limiting T-cell proliferation and survival, whereas liver X receptor beta depletion enhanced the activity of CAR T cells in solid-tumor models 75. These opposing observations have not been fully reconciled and may reflect differences in tumor type, T cell state, spatial localization, or the cholesterol pool measured.

Together, these studies support the view that lipid quality and cholesterol homeostasis shape the membrane organization, metabolic fitness, and signaling competence of CD8^+^ T cells. Excess cholesterol can reinforce exhaustion, whereas excessive esterification or impaired cholesterol supply can weaken TCR signaling, proliferation, and survival. ACAT1 inhibition improved PD-1 blockade in melanoma models, while manipulation of oxysterol-responsive cholesterol regulation enhanced chimeric antigen receptor T cell activity. Whether SCD1 inhibition provides comparable immunotherapy benefit across tumor types remains unresolved. Whereas these mechanisms remodel T cells through intracellular lipid composition and membrane organization, extracellular metabolites and ions provide an additional layer of suppression by engaging inhibitory receptors and perturbing signal transduction.

## Microenvironmental chemical constraints: purinergic signaling, epigenetic encoding, and ionic stress

Beyond competition for glucose, amino acids, and lipids, the tumor microenvironment imposes additional constraints through locally accumulated signaling molecules and altered ionic conditions. These chemical changes act through distinct mechanisms rather than serving primarily as nutrients. Extracellular adenosine activates inhibitory purinergic receptors, lactate can encode glycolytic conditions into cell-state-specific chromatin programs, and potassium released from necrotic cells perturbs T-cell signaling and differentiation. Together, these mechanisms convert local metabolic imbalance and tissue damage into receptor-mediated inhibition, transcriptional regulation, and ionic restraint.

### Adenosine accumulation: A2A receptor signaling and pyrimidine nucleotide stress

Extracellular adenosine converts tissue damage, hypoxia, and metabolic stress into immunosuppressive signals within the tumor microenvironment. Extracellular adenosine triphosphate and adenosine diphosphate released from stressed, dying, or activated cells are hydrolyzed to adenosine monophosphate by ectonucleoside triphosphate diphosphohydrolase 1 (CD39), after which ecto-5′-nucleotidase (CD73) converts adenosine monophosphate into adenosine. Because these enzymes can be expressed by malignant, stromal, endothelial, and immune cells, adenosine production need not be tumor-cell autonomous 76, 77. Hypoxia can further promote adenosine accumulation through tumor-specific metabolic programs. In hepatocellular carcinoma models, hypoxia-inducible factor 1 induced MAX interactor 1, which repressed adenosine kinase, while directly increasing equilibrative nucleoside transporter 4 expression. These changes promoted adenosine efflux and reduced the efficacy of immune checkpoint blockade 78.

Adenosine A2A receptor (A2AR) signaling provides a well-defined extracellular route of T-cell suppression. A2AR engagement increases intracellular cyclic adenosine monophosphate and activates protein kinase A, reducing TCR signaling, cytokine production, and cytotoxic activity. Genetic deletion or pharmacological inhibition of A2AR enhanced antitumor T-cell responses in mouse tumor models 79. Complementary signaling studies in Jurkat and normal T cells showed that protein kinase A activated C-terminal Src kinase (Csk), which inhibited lymphocyte-specific protein tyrosine kinase (Lck) and reduced CD3ζ phosphorylation 80. Together, these tumor-model and cell-signaling studies provide a molecular explanation for A2AR-mediated inhibition of proximal TCR signaling, while indicating that the receptor-level and downstream evidence was obtained from complementary experimental systems.

Adenosine can also suppress activated T cells independently of cell-surface receptor signaling. Activated human T cells upregulated equilibrative nucleoside transporter 1 (ENT1) and internalized extracellular adenosine, which inhibited *de novo* pyrimidine nucleotide synthesis and impaired proliferation, viability, and effector function. Pharmacological ENT1 inhibition restored intracellular pyrimidine pools, enhanced memory T cell-mediated tumor killing, and increased the *ex vivo* expansion of functional human tumor-infiltrating lymphocytes. In a humanized triple-negative breast cancer model, ENT1 inhibition also improved tumor control when combined with programmed cell death protein 1 blockade 81. ENT1-mediated adenosine uptake therefore represents a distinct intracellular metabolic checkpoint rather than a downstream component of A2AR signaling.

Together, these studies identify two mechanistically distinct routes of adenosine-mediated immune suppression: extracellular A2AR signaling inhibits proximal T-cell activation, whereas ENT1-dependent uptake disrupts intracellular pyrimidine metabolism. Their relative importance is likely to depend on the cellular sources of adenosine, local oxygenation, T-cell activation state, and transporter expression. In selected preclinical models, interventions targeting the hypoxia-adenosine axis or ENT1 improved the efficacy of immune checkpoint blockade, indicating that inhibition of adenosine production, receptor antagonism, and restriction of cellular uptake are mechanistically complementary rather than interchangeable strategies. Whereas adenosine acts through purinergic signaling and intracellular nucleotide stress, lactate can encode metabolic conditions into longer-lasting transcriptional programs through histone lactylation.

### Lactate-derived histone lactylation: cell-state-specific epigenetic encoding

Histone lactylation converts cellular metabolic conditions into transcriptional programs, but its immunological consequences depend on the responding cell type and differentiation state. Histone lysine lactylation was identified as a lactate-derived chromatin modification in human and mouse cells. In bacterially challenged macrophages, its delayed accumulation was associated with the induction of homeostatic and wound-healing genes, including Arg1 82. This study established the principle that glycolysis-derived lactate can regulate gene expression through chromatin modification, although it did not examine the tumor microenvironment.

Tumor studies subsequently identified lineage-specific immunosuppressive programs in myeloid cells. In glioblastoma, monocyte-derived macrophages displayed greater glucose uptake and intracellular lactate accumulation than resident microglia. The protein kinase R-like endoplasmic reticulum kinase (PERK)-activating transcription factor 4 (ATF4) pathway increased glucose transporter 1 expression and histone lactylation, thereby promoting interleukin-10 (IL-10)-dependent suppression of T-cell activity. Disruption of this pathway reduced macrophage immunosuppression and improved immunotherapy efficacy in the corresponding models 83. In brain tumor-infiltrating neutrophils, hypoxia similarly induced a highly glycolytic CD71-expressing subset in which histone lactylation promoted ARG1 expression and T-cell suppression. Targeting this program delayed tumor progression and increased immunotherapy sensitivity 84. These findings support distinct IL-10- and ARG1-associated lactylation programs in tumor-associated macrophages and neutrophils, although both were defined primarily in brain tumor models.

Histone lactylation is not uniformly immunosuppressive. In human and mouse CD8^+^ T cells, histone H3 lysine 18 lactylation marked activation-associated genes, whereas histone H3 lysine 9 lactylation showed broader enrichment across naive, activated, and memory states. Both marks were minimally enriched in terminally exhausted CD8^+^ T cells. Experimental manipulation of the metabolic and epigenetic pathways controlling these marks altered effector-gene expression, cytokine production, tumor-cell killing, and antitumor activity in preclinical models 85. Thus, the functional direction of histone lactylation depends on immune-cell lineage, the modified lysine site, the metabolic source of lactate, and differentiation state.

Histone lactylation should therefore be viewed as a cell-state-specific transcriptional mechanism rather than a uniformly suppressive consequence of lactate accumulation. Selective disruption of immunosuppressive myeloid programs may be beneficial, whereas global inhibition may also impair lactylation-dependent CD8^+^ T-cell functions. Whereas lactylation translates metabolic conditions into chromatin regulation, extracellular potassium constrains T cell activity more directly by disrupting ionic homeostasis and intracellular signaling.

### Potassium accumulation: ionic immune suppression and checkpoint resistance

Potassium accumulation in necrotic tumors can constrain ICB by suppressing T cell effector signaling and reinforcing immunosuppressive myeloid metabolism. Necrotic tumor cells release intracellular potassium into the interstitial fluid, thereby increasing intracellular potassium in tumor-reactive T cells. In mouse and human T cell systems, elevated potassium inhibited TCR-induced Akt-mTOR signaling and effector cytokine production through a protein phosphatase 2A-dependent mechanism. Enforced expression of the voltage-gated potassium channel Kv1.3 increased potassium efflux, restored T cell effector function, and improved tumor control in mouse melanoma models 86. Because checkpoint blockade relies on the recovery of TCR signaling and cytotoxic activity, potassium-rich tumor regions may remain poorly responsive despite inhibitory-receptor blockade.

High extracellular potassium can also maintain myeloid programs that oppose immunotherapy. In tumor-associated macrophages, the inwardly rectifying potassium channel Kir2.1 supported glutamine uptake and oxidative metabolism under potassium-rich conditions. Kir2.1 disruption shifted macrophages toward a more antitumor state and reduced tumor growth in preclinical models 87. In myeloid-derived suppressor cells, high potassium activated a Kir4.1-fatty acid-binding protein 3 program that increased fatty-acid uptake, oxidation, and T-cell suppression. Kir4.1 inhibition reduced immunosuppressive activity and improved PD-1 blockade in preclinical models 88. Potassium accumulation can therefore limit immunotherapy through both direct T cell inhibition and metabolic stabilization of suppressive myeloid populations.

Elevated potassium nevertheless produces a treatment-dependent trade-off. By restricting glucose and lipid uptake, it induced functional caloric restriction, autophagy, and reduced nucleocytosolic acetyl-coenzyme A in T cells. The associated decrease in histone acetylation limited terminal effector and exhaustion programs while preserving less-differentiated, persistent properties. T cells conditioned under high-potassium conditions consequently showed weaker immediate effector activity but improved expansion, persistence, and tumor clearance after adoptive transfer 89. This does not imply that sustained intratumoral potassium supports effective immunity. Instead, controlled metabolic conditioning may be exploitable during cell-therapy preparation.

Overall, extracellular potassium is a cell-type- and treatment-context-dependent contributor to immunotherapy resistance. Selective enhancement of potassium efflux in antitumor T cells or inhibition of myeloid Kir channels may therefore be more effective than nonselective manipulation of systemic potassium.

Together, the Local Resource Axis captures how extracellular nutrient restriction, enzymatic depletion, lipid dysregulation, and the accumulation of bioactive metabolites and ions constrain antitumor immunity. Although these pressures act through distinct molecular routes and over different timescales, they converge on impaired T-cell signaling, biosynthesis, redox homeostasis, and functional persistence. At the intracellular and intercellular levels, sustained metabolic stress can disrupt mitochondrial and endoplasmic reticulum homeostasis, whereas mitochondrial transfer between immune and malignant cells can redistribute metabolic capacity and organelle damage.

## Organelle axis: intracellular organelle dysfunction and intercellular mitochondrial redistribution

Whereas the Local Resource Axis describes extracellular metabolic constraints, the Organelle Axis addresses how immune-cell fitness is shaped by intracellular organelle dysfunction and intercellular mitochondrial redistribution. Three mechanistically distinct processes are considered: impaired mitochondrial biogenesis and quality control, direction- and cargo-dependent mitochondrial transfer, and endoplasmic reticulum stress with disrupted mitochondria-endoplasmic reticulum coordination. Together, these processes reduce T cell metabolic reserve, redistribute mitochondrial fitness and damage between cells, and weaken both T-cell recovery and immune priming.

## Mitochondrial dysregulation: reduced metabolic reserve and limited T-cell recovery

Effective ICB requires tumor-reactive T cells to recover proliferative capacity, persistence, and sustained cytotoxic function after inhibitory signaling is relieved. Mitochondrial dysfunction can constrain this recovery through three related processes: insufficient functional mitochondrial capacity, defective clearance of damaged mitochondria, and mitochondrial DNA (mtDNA)-associated senescence signaling.

### Mitochondrial architecture and biogenesis: metabolic reserve for persistence and cytotoxicity

Mitochondrial architecture and biogenesis determine whether T cells can meet the energetic demands of expansion and repeated effector activity. In functional memory CD8^+^ T cells, optic atrophy 1 (OPA1)-dependent mitochondrial fusion and cristae organization promote electron transport chain (ETC) assembly and OXPHOS 90. In tumors, repression of peroxisome proliferator-activated receptor gamma coactivator 1-alpha (PGC-1α)-dependent mitochondrial biogenesis reduces mitochondrial mass and contributes to metabolic insufficiency in CD8^+^ tumor-infiltrating lymphocytes (TILs) 91.

These defects affect complementary aspects of T-cell function. Impaired OXPHOS limits the self-renewal of T cells exposed to persistent antigen, whereas continuous stimulation under hypoxia increases mitochondrial reactive oxygen species (ROS) and accelerates exhaustion 92, 93. Mitochondrial translation is also required for the sustained replenishment of perforin and granzymes during serial target-cell killing 94. Together, these findings show that mitochondrial capacity supports T cell self-renewal, persistence, and sustained cytotoxicity. Its loss may therefore restrict the functional recovery required after PD-1 blockade.

### Defective mitophagy: reinforcement of terminal exhaustion

Whereas impaired biogenesis limits the generation of functional mitochondria, defective mitophagy allows depolarized and dysfunctional mitochondria to accumulate in CD8^+^ TILs 95. This failure of mitochondrial quality control promotes transcriptional and epigenetic programs associated with terminal exhaustion, reducing the functional plasticity available for subsequent T-cell recovery.

Mitochondrial restoration experiments support the therapeutic relevance of this mechanism. Nicotinamide riboside improved mitochondrial fitness and TIL function and increased responsiveness to PD-1 blockade in preclinical tumor models 95. Ubiquitin-specific peptidase 30 (USP30), a mitochondrial deubiquitinase that restricts mitophagy, provides a more selective molecular target. USP30 is upregulated in exhausted CD8^+^ T cells, whereas its genetic deletion or pharmacological inhibition enhances mitophagy, restores mitochondrial function, and improves antitumor activity 96. Defective mitophagy can therefore stabilize a dysfunctional mitochondrial state that reinforces terminal exhaustion, whereas restoration of mitochondrial quality control may recover the cellular fitness required for reinvigoration.

### Mitochondrial DNA stress: a potential senescence-associated barrier to recovery

Damaged mitochondria may impose a more persistent functional barrier through cytosolic DNA sensing and senescence-associated signaling. In mitochondrial transcription factor A (TFAM)-deficient models, mtDNA stress activates cyclic GMP-AMP synthase (cGAS)-stimulator of interferon genes (STING) signaling 97. Separately, cGAS-mediated recognition of cytosolic chromatin fragments has been shown to promote cell-cycle arrest and the senescence-associated secretory phenotype (SASP) 98.

Evidence from CD8^+^ T cells further connects mitochondrial homeostasis with STING-associated senescence. Nicotinamide adenine dinucleotide (NAD^+^) depletion is associated with mitochondrial dysfunction, cytosolic mtDNA accumulation, STING activation, and cellular senescence, whereas NAD^+^ restoration attenuates these changes 99. Because senescent T cells are growth arrested, this pathway may restrict the proliferative recovery required after PD-1 blockade. However, the complete sequence from defective mitophagy to cGAS-STING-dependent TIL senescence and ICB resistance has not been demonstrated in a single tumor model and is therefore best viewed as a plausible extension of mitochondrial dysfunction rather than an established universal mechanism.

Collectively, mitochondrial dysregulation can constrain T-cell recovery at three functional levels. Reduced mitochondrial biogenesis limits the metabolic reserve required for expansion and persistence, defective mitophagy reinforces terminal exhaustion, and mtDNA-associated stress may impose a senescence-related barrier to proliferation. These findings indicate that relief of inhibitory receptor signaling may be insufficient when the mitochondrial machinery required for T-cell recovery has already been compromised.

## Intercellular mitochondrial transfer: direction- and cargo-dependent redistribution of metabolic fitness

Intercellular mitochondrial transfer can support or suppress antitumor immunity depending on the identities of the donor and recipient cells, the functional state of the transferred mitochondria, and the molecular cargo that accompanies them. Bone marrow stromal cells (BMSCs) can transfer functional mitochondria to CD8^+^ T cells through Talin 2-dependent tunneling nanotubes (TNTs), increasing mitochondrial respiration, expansion, persistence, and subsequent antitumor efficacy 100. These findings demonstrate the therapeutic potential of controlled mitochondrial delivery to restore T cell metabolic fitness.

By contrast, immune-to-tumor mitochondrial transfer can benefit malignant cells. Cancer cell-derived TNTs enable tumor cells to acquire mitochondria from T cells and other immune cells, reducing mitochondrial content and respiratory capacity in donor cells while increasing metabolic fitness in recipient tumor cells 101. More recent evidence indicates that immune-derived mitochondria can integrate into the endogenous mitochondrial network of tumor cells, activate mtDNA-cGAS-STING signaling, and induce type I interferon-associated programs that promote lymph-node metastasis 102. Immune-to-tumor transfer can therefore simultaneously weaken immune-cell fitness and expand the metabolic and signaling capacity of malignant cells.

A distinct pathological outcome arises when mitochondria move from tumor cells into immune cells. Tumor cells can transfer mitochondria carrying pathogenic mtDNA mutations, together with molecular factors that interfere with mitochondrial clearance, into TILs 103. Recipient T cells subsequently develop mitochondrial dysfunction, cellular senescence, defective memory formation, and reduced responsiveness to immune checkpoint inhibitors 103. Tumor-to-T cell transfer thus directly links the acquisition of pathogenic mitochondrial states to impaired T cell reinvigoration and checkpoint responsiveness.

These findings establish intercellular mitochondrial transfer as a direction- and cargo-dependent process rather than a uniformly supportive or suppressive phenomenon. Immune-to-tumor transfer redistributes metabolic capacity toward malignant cells, whereas tumor-to-T cell transfer imposes dysfunctional mitochondrial states on antitumor lymphocytes. By contrast, controlled stromal-to-T cell transfer can enhance T cell metabolic fitness. These routes therefore represent distinct forms of organelle redistribution rather than a single linear mechanism of “mitochondrial theft.” Therapeutic strategies will need to distinguish among them by limiting mitochondrial acquisition by tumor cells, preventing the transfer or persistence of pathogenic tumor-derived mitochondria in T cells, and selectively harnessing functional mitochondrial delivery for cellular immunotherapy.

## Endoplasmic reticulum-mitochondrial dysregulation: impaired organelle coupling and stress-response signaling

Effective ICB depends not only on the release of inhibitory receptor signaling but also on the cellular machinery required to convert that release into T cell expansion and sustained antitumor activity. Endoplasmic reticulum-mitochondrial dysregulation can compromise this machinery through two mechanistically distinct processes: disruption of mitochondria-endoplasmic reticulum contact site-dependent metabolic coordination in CD8^+^ T cells and maladaptive unfolded protein response (UPR) signaling in T cells and dendritic cells. These processes converge on two requirements for durable antitumor immunity: the metabolic recovery of tumor-reactive T cells and the immunostimulatory support provided by dendritic cells.

### Mitochondria-endoplasmic reticulum coupling: metabolic coordination of T cell recall and tumor fitness

Mitochondria-endoplasmic reticulum contact sites (MERCs) organize signaling and substrate utilization during T cell metabolic activation. In memory CD8^+^ T cells, MERCs recruit the mechanistic target of rapamycin complex 2 (mTORC2)-Akt signaling axis to mitochondria, inhibit glycogen synthase kinase 3 beta (GSK3β), and promote hexokinase I-dependent pyruvate oxidation during rapid recall responses 104. These findings establish MERCs as physiological immunometabolic platforms that enable previously activated T cells to rapidly resume effector function.

Tumor-specific evidence further links MERC integrity to the metabolic competence of CD8^+^ TILs. Mitofusin 2 (MFN2) interacts with sarco/endoplasmic reticulum Ca²^+^-ATPase 2 (SERCA2) to maintain mitochondria-endoplasmic reticulum contact, calcium homeostasis, mitochondrial metabolism, and antitumor function in CD8^+^ TILs. Increasing MFN2 expression restored T-cell metabolic fitness and improved therapeutic efficacy in preclinical tumor models 105.

Together, the physiological and tumor-model evidence positions MERCs as structural platforms that connect organelle organization with the metabolic flexibility required for both recall responses and tumor control. Disruption of this coupling may therefore leave T cells unable to generate the metabolic output required for expansion and effector activity even after PD-1-mediated inhibition is relieved.

### Unfolded protein response signaling: cell-type-specific disruption of T-cell fitness and immune priming

Chronic endoplasmic reticulum stress activates UPR pathways whose functional consequences depend on both the signaling branch and the immune-cell compartment involved. In ovarian cancer models, activation of the inositol-requiring enzyme 1 alpha (IRE1α)-X-box binding protein 1 (XBP1) pathway in T cells restricts glutamine-supported mitochondrial respiration and impairs antitumor function 106.

In tumor-associated dendritic cells (DCs), constitutive XBP1 signaling promotes abnormal lipid accumulation and impairs immunostimulatory capacity, thereby weakening support for antitumor T-cell responses 107. The same UPR branch can therefore disrupt antitumor immunity through distinct metabolic effects in T cells and DCs.

A separate UPR branch links endoplasmic reticulum stress more directly to mitochondrial oxidative injury in TILs. PERK and its downstream effector endoplasmic reticulum oxidoreductase 1 alpha (ERO1α) increase mitochondrial reactive oxygen species in tumor antigen-specific CD8^+^ TILs 108. PERK inhibition reduces mitochondrial oxidative stress, improves T cell viability, and enhances antitumor responses, including responses to combined PD-1 blockade, in preclinical models 108.

Together, these branch- and cell-type-specific lesions converge by weakening T cell metabolic fitness and the dendritic-cell support required for effective antitumor immunity.

Collectively, the Organelle Axis shows that ICB efficacy depends on organelle competence as well as inhibitory receptor signaling. Defective mitochondrial homeostasis reduces the metabolic reserve of T cells, pathological mitochondrial transfer redistributes organelle fitness and damage between immune and malignant cells, and endoplasmic reticulum-mitochondrial dysregulation weakens both T-cell function and dendritic-cell support. Most therapeutic evidence remains preclinical.

The next section shifts from cellular competence to tissue access, focusing on the stromal, vascular, and organ-specific barriers that constrain T-cell entry and function.

## Spatial axis: tissue architecture and organ-specific immune-metabolic niches

The Spatial Axis describes how metabolic and immune pressures are organized by tissue architecture and anatomical context rather than simply by the identity of individual metabolites or stress pathways. At the local level, stromal and vascular compartments regulate tumor-cell metabolic support, perfusion, therapeutic penetration, and lymphocyte entry. At the organ level, barrier architecture, resident cell populations, and physiological metabolism further reshape these constraints, generating site-specific patterns of tumor adaptation, immune dysfunction, and resistance to immunotherapy. Figure 2 summarizes this spatial logic by integrating the shared stromal-vascular gate with brain, liver, bone, and bone marrow niches as anatomically distinct immune-metabolic filters that shape ICB responsiveness.

## Stromal remodeling: metabolic support, vascular compression, and immune exclusion

Stromal cells contribute to treatment resistance through two complementary functions: they support tumor-cell adaptation to nutrient stress and remodel tissue architecture in ways that restrict vascular and immune access. Cancer-associated fibroblasts (CAFs), a major stromal population in many solid tumors, can therefore influence both the metabolic resources available to malignant cells and the spatial conditions required for effective antitumor immunity.

### Autophagy-dependent stromal support: tumor metabolic adaptation under nutrient limitation

Autophagy-associated stromal programs support tumor-cell adaptation to nutrient limitation through both fibroblast metabolic reprogramming and the release of alternative substrates. In stromal fibroblasts, loss of sequestosome 1 (p62/SQSTM1) impairs mechanistic target of rapamycin complex 1 (mTORC1) signaling and establishes a pro-tumorigenic metabolic state 109. This finding defines a regulatory level of stromal metabolic support that is distinct from the specific metabolite-transfer pathways described below. More directly, pancreatic stellate cells in PDAC use autophagy to release alanine, which tumor cells incorporate into the tricarboxylic acid (TCA) cycle, thereby preserving glucose-derived carbon for serine and glycine biosynthesis 110. Under glutamine limitation, CAFs can also use nuclear fragile X mental retardation-interacting protein 1 (NUFIP1)-dependent ribophagy to release nucleosides that sustain MYC-dependent glucose utilization and tumor growth 111. Together, these studies distinguish two complementary levels of autophagy-associated stromal support: metabolic reprogramming establishes a tumor-supportive fibroblast state, whereas autophagy-dependent substrate-release programs provide alternative nutrients under distinct nutrient-limited conditions.

### Desmoplastic remodeling: vascular compression and immune exclusion

Desmoplastic remodeling can restrict treatment responses through both abnormal tissue mechanics and an immunosuppressive stromal state. In PDAC, accumulation of hyaluronan (HA) generates abnormal tissue mechanics that compress intratumoral vessels and impair perfusion and drug delivery. In autochthonous PDAC models, enzymatic depletion of HA with PEGylated recombinant human hyaluronidase PH20 (PEGPH20) reduced interstitial fluid pressure, re-expanded the microvasculature, and improved the intratumoral delivery and efficacy of gemcitabine 112. Subsequent studies extended this physical-access model by showing that PEGPH20-mediated HA depletion reoxygenated pancreatic tumor xenografts 113 and increased anti-PD-L1 antibody uptake and lymphocyte accumulation in HA-rich breast tumor models 114. Together, these findings identify HA-rich stroma as a spatial barrier that restricts vascular supply, therapeutic penetration, and immune-cell access, whereas HA depletion can restore tissue conditions that are more permissive to antitumor immunity and checkpoint therapy.

Beyond HA-mediated vascular compression, tumor-cell focal adhesion kinase (FAK) signaling contributes to the maintenance of a fibrotic and immunosuppressive stromal state. In human PDAC tissues, elevated FAK activity was associated with greater fibrosis and reduced CD8^+^ T-cell infiltration. In mouse PDAC models, pharmacological FAK inhibition reduced collagen deposition, fibroblast activation protein-positive stromal cells, and immunosuppressive myeloid-cell accumulation, and sensitized otherwise resistant tumors to adoptive T cell therapy and PD-1 blockade 115. Thus, desmoplasia can constrain therapy through two distinct but convergent routes: HA-rich matrix compresses intratumoral vessels and restricts tissue access, whereas tumor-cell FAK signaling maintains a fibrotic, immunosuppressive niche that limits T-cell infiltration and checkpoint responsiveness.

Overall, stromal remodeling couples metabolic support for tumor cells with physical and immunological barriers to perfusion, therapeutic penetration, and T cell access.

## Vascular dysfunction and remodeling: immune exclusion and lymphocyte recruitment

Whereas stromal remodeling shapes access within the tumor interstitium, tumor endothelial cells (TECs) regulate entry and survival at the vascular interface. Endothelial hyperglycolysis can sustain pathological angiogenesis and metabolite-mediated immune suppression, endothelial Fas ligand (FasL) can selectively eliminate activated CD8^+^ T cells, and vascular normalization or venular specialization can restore lymphocyte recruitment. These experimentally distinct mechanisms converge on the number and functional state of tumor-reactive T cells available for reinvigoration by ICB.

### Endothelial hyperglycolysis: vascular instability and metabolite-mediated immune suppression

Angiogenic endothelial cells rely heavily on glycolysis driven by 6-phosphofructo-2-kinase/fructose-2,6-bisphosphatase 3 (PFKFB3) to support endothelial migration, proliferation, and vessel sprouting 116. Partial and transient reduction of PFKFB3 activity is sufficient to suppress pathological angiogenesis without completely ablating the vasculature 117. In tumors, this hyperglycolytic program has both structural and immunometabolic consequences. Structurally, partial genetic or pharmacological inhibition of endothelial PFKFB3 reduces vascular endothelial cadherin (VE-cadherin) endocytosis, strengthens endothelial junctions, and increases pericyte adhesion, thereby promoting vessel normalization and improving therapeutic delivery 118. Metabolically, independent evidence from colorectal cancer models showed that hyperglycolytic tumor endothelial cells release lactate, whereas inhibition of endothelial GAPDH reduced lactate production, promoted vascular normalization and effector immune-cell infiltration, and enhanced the efficacy of PD-1 blockade 119. Together, these findings indicate that endothelial hyperglycolysis can constrain antitumor immunity through both structural destabilization of tumor vessels and metabolite-mediated remodeling of the immune microenvironment.

### Endothelial Fas ligand expression: selective T-cell apoptosis and immune exclusion

Beyond glycolysis-associated vascular abnormalities, TECs can acquire an immunosuppressive surface phenotype that selectively restricts effector T-cell accumulation. Tumor-derived vascular endothelial growth factor A (VEGF-A) can induce endothelial production of prostaglandin E2 (PGE2), thereby suppressing T-cell proliferation and cytokine production 120. More broadly, tumor-derived cyclooxygenase (COX)-dependent PGE2 promotes immune evasion 121 and disrupts the natural killer cell-conventional type 1 dendritic cell recruitment axis 122. At the vascular interface, VEGF-A, IL-10, and PGE2 cooperatively induce Fas ligand (FasL) expression on TECs 123. Endothelial FasL selectively triggers apoptosis in activated CD8^+^ T cells, whereas regulatory T cells are relatively protected by higher expression of cellular FLICE-inhibitory protein (c-FLIP), thereby shifting the intratumoral CD8^+^ T cell/regulatory T cell balance toward immune tolerance 123. In mouse tumor models, pharmacological suppression of COX/PGE2 and VEGF-A reduced endothelial FasL expression and increased CD8^+^ T-cell infiltration, whereas direct FasL blockade enhanced the efficacy of adoptive T cell therapy 123. Together, these findings identify endothelial FasL as a selective vascular death signal that limits effector T-cell accumulation and weakens T-cell-based antitumor therapy.

### Vascular remodeling: lymphocyte recruitment and improved immunotherapy responsiveness

Immune checkpoint blockade can reinvigorate tumor-reactive T cells, but its efficacy remains constrained when these cells cannot efficiently cross the tumor vasculature. Vascular normalization can complement checkpoint inhibition by restoring endothelial conditions that support immune-cell entry. In preclinical tumor models, dual blockade of angiopoietin-2 (Ang2) and VEGF-A with the Ang2/VEGF-A (A2V) bispecific antibody normalized the remaining tumor vessels and promoted the extravasation and perivascular accumulation of activated, IFN-γ-expressing CD8^+^ cytotoxic T lymphocytes. The addition of PD-1 blockade further improved tumor control, indicating that vascular normalization and checkpoint reinvigoration can act through complementary mechanisms 124.

Beyond restoring conventional vessel function, vascular remodeling can generate specialized routes for lymphocyte recruitment. Combined antiangiogenic and anti-programmed death-ligand 1 (PD-L1) therapy induced the formation of high endothelial venules (HEVs) in tumor models 125. Subsequent work showed that CD8^+^ T cell- and natural killer cell-derived signals activate lymphotoxin beta receptor (LTβR) signaling in post-capillary venules, promoting their transition into HEVs and creating vascular niches enriched for T cell factor 1 (TCF1)-positive progenitor-like CD8^+^ T cells 126. Tumor-associated high endothelial venules (TA-HEVs) were identified as major sites of lymphocyte arrest and extravasation during combined PD-1 and CTLA-4 blockade, and their abundance predicted improved response and survival in patients with metastatic melanoma 127. More recently, endothelial expression of chicken ovalbumin upstream promoter-transcription factor II (COUP-TFII) was shown to induce post-capillary venular programs, enhance T-cell recruitment, and sensitize breast and pancreatic tumor models to immune checkpoint blockade and adoptive T cell therapy 128. Normalization restores the function of existing tumor vessels, whereas high endothelial venule (HEV) differentiation and venular reprogramming establish specialized routes and niches for lymphocyte recruitment. By increasing the access and intratumoral accumulation of tumor-reactive T cells, these forms of vascular remodeling can alleviate a major spatial component of resistance to immune checkpoint blockade.

More broadly, stromal and vascular remodeling jointly determine whether tumor-reactive T cells can reach, enter, and persist within tumor tissue. These shared spatial constraints are further reshaped by the intrinsic barrier architecture, resident cell populations, and metabolic physiology of individual organs.

## Organ-specific metabolic niches: tissue physiology and site-specific immune resistance

Organ-specific barrier architecture, nutrient availability, resident cell populations, and homeostatic immune programs shape the metabolic conditions encountered by malignant and immune cells. Tumors can exploit these pre-existing tissue properties through distinct combinations of metabolic adaptation, stromal remodeling, and immune reprogramming, thereby generating site-specific constraints on antitumor immunity and immune checkpoint responsiveness 129.

### Brain: constrained nutrient availability, metabolic autonomy, and metabolite-mediated immune suppression

The cerebral microenvironment imposes a distinct set of metabolic constraints through selective blood-brain barrier (BBB) transport and the specialized nutrient demands of neural tissue. These conditions shape both primary gliomas and brain metastases (BrM), selecting for malignant cells that can synthesize limiting metabolites or exploit alternative carbon sources. In parallel, glioma-derived metabolites can directly suppress local myeloid and lymphoid immunity, reducing the pool of functional tumor-reactive T cells available for reinvigoration by immune checkpoint blockade.

### Selective nutrient availability: biosynthetic autonomy in brain metastasis

Relative restriction of extracellular lipids can create a site-specific requirement for *de novo* lipid synthesis. In breast cancer models, tumor cells growing in the brain displayed increased fatty acid synthesis compared with extracranial lesions, and genetic or pharmacological inhibition of FASN preferentially impaired brain-metastatic growth 130. Limited serine and glycine availability can impose a related biosynthetic requirement. BrM cells with elevated 3-phosphoglycerate dehydrogenase (PHGDH) activity maintained *de novo* serine synthesis and nucleotide production, whereas PHGDH inhibition selectively reduced tumor growth in the brain 131. The strength of this dependency varies across tumor models, indicating that the cerebral nutrient environment interacts with tumor-intrinsic metabolic capacity rather than imposing a uniform program 132.

This metabolic asymmetry may also have immunological consequences. Efficient effector T cell expansion requires extracellular serine to sustain one-carbon metabolism and nucleotide synthesis 133. Tumor cells capable of maintaining lipid and serine biosynthesis are therefore better positioned to withstand the cerebral nutrient environment than infiltrating lymphocytes that remain more dependent on extracellular substrate availability. Thus, biosynthetic autonomy supports brain colonization while potentially narrowing the proliferative reserve of local antitumor T cells.

### ACSS2-dependent acetate utilization: adaptation to nutrient stress

Glioblastoma (GBM) and BrM can also exploit acetate as an alternative carbon source. Isotope-tracing studies in patients demonstrated substantial acetate oxidation in both GBM and brain-metastatic tissues, establishing acetate as a physiologically relevant bioenergetic substrate in human brain tumors 134. Under glucose limitation, acetyl-coenzyme A synthetase 2 (ACSS2) translocates to the nucleus and generates local acetyl-coenzyme A pools that support histone acetylation and the transcription of lysosomal and autophagy-related genes, thereby promoting tumor-cell survival during nutrient stress 135.

Acetate can also sustain CD8^+^ T cell histone acetylation, chromatin accessibility, and IFN-γ production under glucose restriction 136. Together, these findings identify acetate metabolism as a stress-adaptation pathway available to both malignant and immune cells. In brain tumors, however, the best-established role of ACSS2-dependent acetate utilization is to confer malignant-cell metabolic flexibility by sustaining bioenergetic and transcriptional programs under nutrient limitation. Beyond adapting to these constraints, glioma cells can actively reshape the local immune environment through the production of bioactive metabolites.

### Glioma-derived metabolites: direct suppression of antitumor immunity

Metabolomic profiling identified accumulation of the tryptophan-derived metabolite quinolinate in GBM. Quinolinate activated N-methyl-D-aspartate receptor (NMDAR)-FOXO1-peroxisome proliferator-activated receptor gamma (PPARγ) signaling in tumor-associated macrophages, promoting a tumor-supportive phenotype, whereas restriction of quinolinate production reduced myeloid immunosuppression in glioma models 137.

A more direct effect on cytotoxic lymphocytes has been demonstrated for spermidine. Spermidine accumulated in the GBM microenvironment and was produced in part by malignant cells. Tumor-derived spermidine increased reactive oxygen species and apoptosis in CD8^+^ T cells while reducing IFN-γ, TNF-α, granzyme B, and perforin production. Suppression of tumor-cell ornithine decarboxylase 1 (ODC1) reduced spermidine abundance, increased intratumoral CD8^+^ T-cell proliferation and accumulation, and prolonged survival in mouse GBM models. In human GBM, elevated ODC1 expression and spermidine levels were associated with reduced CD8^+^ T-cell infiltration and poorer survival 138.

Together, these findings reveal two complementary dimensions of brain-specific metabolic resistance. Restricted nutrient availability selects for tumor cells with enhanced biosynthetic autonomy and alternative-substrate utilization, whereas glioma-derived metabolites directly sustain immunosuppressive macrophage states and impair cytotoxic T-cell fitness. By preserving malignant-cell survival while reducing the abundance and functionality of antitumor effector cells, these mechanisms may create a cerebral metabolic niche that limits the effectiveness of T-cell-based immunotherapy and checkpoint-mediated immune reinvigoration.

### Liver: tolerogenic physiology and metabolite-driven immune dysfunction

The liver is continuously exposed to gut-derived nutrients, microbial products, and circulating antigens through the portal circulation and therefore maintains a physiologically restrained immune environment. Primary liver tumors and liver metastases exploit this tolerogenic setting through etiology-dependent T-cell dysfunction, macrophage-mediated T cell loss, bile acid dysregulation, and differential adaptation to nitrogenous waste. Together, these mechanisms reduce the abundance or functional competence of tumor-reactive lymphocytes and constrain immune checkpoint therapy.

### Steatohepatitis-associated T-cell dysfunction: etiology-dependent checkpoint resistance

The underlying etiology of HCC can shape the functional state of the hepatic T-cell compartment. In preclinical models of non-alcoholic steatohepatitis-associated HCC (NASH-HCC), PD-1 blockade expanded activated CD8^+^PD-1^+^ T cells but failed to induce tumor regression. These cells displayed tissue-resident, dysfunctional, and inflammatory features, including expression of CXCR6, TOX, and tumor necrosis factor. In preventive settings, PD-1 blockade aggravated liver injury and increased HCC development through CD8^+^ T cell- and tumor necrosis factor-dependent mechanisms 139. Related CD8^+^PD-1^+^ T cell states were detected in human fatty liver disease, while the reported clinical analyses suggested reduced immunotherapy benefit in non-viral and NASH-associated HCC 139. Thus, PD-1 expression in steatohepatitis can mark a tissue-damaging immune population rather than a tumor-reactive compartment that is productively reinvigorated by checkpoint blockade.

### Liver metastasis: macrophage-mediated systemic T cell deletion and exhaustion

Liver metastases can suppress antitumor immunity beyond the hepatic lesion. In patients and mouse models, liver metastases were associated with reduced systemic T cell abundance and impaired responses at extrahepatic tumor sites. Mechanistically, circulating activated Fas^+^CD8^+^ T cells were recruited to the liver and underwent apoptosis after interacting with Fas ligand-positive monocyte-derived macrophages. Liver-directed radiotherapy depleted these suppressive macrophages, preserved hepatic T-cell survival, and restored systemic immunotherapy activity in preclinical models 140.

A complementary mechanism involves secreted phosphoprotein 1-positive (SPP1^+^) macrophages. In colorectal cancer liver metastases, tumor-reactive CD8^+^ T cells were enriched in dysfunctional states and spatially associated with SPP1^+^ macrophages in profibrotic regions. SPP1-CD44 signaling promoted tumor-specific CD8^+^ T cell exhaustion, linking macrophage polarization and fibrotic remodeling to liver-specific immune dysfunction 141. Thus, hepatic macrophages can restrict immunotherapy through both deletion of activated T cells and sustained impairment of the tumor-reactive cells that remain.

### Bile acid composition: impaired lymphocyte recruitment and direct T cell stress

The enterohepatic circulation creates a liver-specific route through which microbial and hepatic metabolism regulate antitumor immunity. Gut bacterial conversion of primary into secondary bile acids reduces C-X-C motif chemokine ligand 16 (CXCL16) expression by liver sinusoidal endothelial cells and limits the recruitment of C-X-C chemokine receptor 6-positive natural killer T cells. Manipulating microbial bile acid conversion increased hepatic natural killer T-cell accumulation and suppressed primary and metastatic liver tumors in mouse models 142.

Bile acids also act directly on tumor-reactive T cells. In liver cancer models, deletion of the hepatic bile acid-conjugating enzyme bile acid-CoA acid N-acyltransferase (BAAT) reduced conjugated bile acids, enhanced tumor-specific T-cell responses, inhibited tumor growth, and sensitized tumors to PD-1 blockade 143. Different bile acid species produced distinct forms of T cell stress: selected primary or conjugated bile acids increased oxidative stress, whereas lithocholic acid impaired T-cell function through endoplasmic reticulum stress. Ursodeoxycholic acid partially restored T-cell fitness and immunotherapy responsiveness 143. Bile acid composition therefore regulates hepatic immunity through both lymphocyte recruitment and direct metabolic effects on intratumoral T cells.

### Ammonia accumulation: effector T cell loss and Treg metabolic adaptation

Ammonia is generated by amino-acid catabolism and can accumulate when tumor metabolism exceeds local nitrogen-disposal capacity. In colorectal cancer models, elevated microenvironmental ammonia reduced T-cell proliferation and cytokine production and promoted exhaustion, whereas enhancing ammonia clearance restored T cell activity and improved immunotherapy response 144.

Recent spatial analyses of human and mouse HCC identified ammonia-rich tumor regions characterized by impaired conventional T-cell survival and preferential enrichment of regulatory T cells (Tregs) 145. Tregs adapted by increasing argininosuccinate lyase-dependent urea-cycle activity to detoxify ammonia and by converting nitrogen into spermine through forkhead box P3-regulated spermine synthase. Spermine enhanced peroxisome proliferator-activated receptor gamma-dependent oxidative phosphorylation and suppressive Treg function. Reducing tumor ammonia production limited Treg-mediated suppression and improved the response to PD-1 blockade in HCC models 145. Ammonia-rich niches therefore create a selective metabolic filter in which effector T cells are impaired while Tregs convert nitrogenous waste into a source of metabolic fitness.

Together, these findings show that hepatic immune resistance arises through several organ-linked but mechanistically distinct processes. Steatohepatitis generates dysfunctional inflammatory T cell states, liver metastases engage macrophage programs that delete or exhaust tumor-reactive lymphocytes, bile acids regulate both immune-cell recruitment and T cell stress, and ammonia selectively favors Treg persistence over effector-cell fitness. These mechanisms reduce the functional T cell pool available for checkpoint-mediated reinvigoration and help explain why immunotherapy responsiveness varies with liver disease etiology, metastatic involvement, and local metabolite composition.

### Bone: osteolysis-driven metabolic and immune remodeling

The bone microenvironment is defined by a mineralized collagen matrix that stores growth factors and undergoes continuous remodeling by osteoclasts and osteoblasts. Bone metastases exploit this physiological turnover to release matrix-associated signals and metabolites while reprogramming osteoclasts into regulators of local and systemic antitumor immunity. These processes jointly support skeletal colonization and can reduce responsiveness to immune checkpoint blockade.

### Osteolysis-associated TGF-β release: altered T cell polarization and reduced checkpoint responsiveness

Tumor-induced osteolysis increases the local availability of matrix-derived transforming growth factor beta (TGF-β), linking bone resorption to metastatic expansion and immune dysfunction. The bone matrix stores latent TGF-β, which is released and activated during osteoclast-mediated resorption. In osteolytic metastases, tumor-derived mediators such as parathyroid hormone-related protein (PTHrP) stimulate osteoclast activity, whereas released TGF-β acts on malignant cells to further increase PTHrP production. In breast cancer bone-metastasis models, this reciprocal signaling reinforces osteoclast activation, bone destruction, and tumor growth, forming the established vicious cycle of osteolytic metastasis 146. The same resorptive process also increases the exposure of local immune cells to matrix-derived TGF-β.

In bone lesions, the best-supported immunological consequence of osteolysis-associated TGF-β is altered T cell polarization. In an intraosseous model of castration-resistant prostate cancer, osteoclast-mediated TGF-β release restrained T helper type 1 (Th1) lineage development and biased the intratumoral CD4^+^ T cell response toward a T helper type 17 (Th17) phenotype. Immune checkpoint therapy alone failed to elicit effective antitumor activity in this setting, whereas combined TGF-β and checkpoint blockade increased Th1 responses, promoted clonal expansion of CD8^+^ T cells, induced regression of bone tumors, and improved survival 147. These intervention data support a causal link between osteolysis-associated TGF-β, suppression of Th1-polarized antitumor immunity, and reduced checkpoint responsiveness. In other tumor settings, stromal TGF-β can additionally restrict CD8^+^ T cell penetration, indicating that its immunological consequences depend on the cellular and spatial context 148.

### Osteolysis-derived hydroxyproline: metabolic reinforcement of skeletal colonization

Osteolysis releases not only matrix-associated growth factors but also amino-acid derivatives from the collagen-rich bone matrix. Hydroxyproline (Hyp), a major product of collagen degradation, can be metabolized by proline dehydrogenase 2 (PRODH2), which is elevated in clinical breast cancer bone-metastasis samples 149. PRODH2-dependent Hyp metabolism generates acetyl-coenzyme A and promotes Yin Yang 1 (YY1) acetylation, thereby increasing expression of the ferroptosis-protective transporter subunit solute carrier family 7 member 11 (SLC7A11) and the osteoclastogenic cytokine IL-8. This program simultaneously enhances tumor-cell resistance to ferroptosis and promotes osteoclast differentiation, while PRODH2 inhibition reduces osteolytic progression *in vivo* 149. Thus, degradation of the bone matrix supplies a metabolic substrate that reinforces both malignant-cell survival and further bone resorption through a PRODH2-SLC7A11/IL-8 feedback circuit.

### Osteoclast-centered immune suppression: local and systemic checkpoint resistance

Osteoclasts can directly regulate antitumor immunity in addition to executing bone resorption. During physiological turnover, apoptotic osteoclasts release apoptotic bodies carrying sialic acid-binding immunoglobulin-like lectin 15 (Siglec-15). Siglec-15 engages sialylated Toll-like receptor 2 on naïve CD8^+^ T cells and suppresses their activation, whereas Siglec-15 neutralization reduces secondary metastasis in breast cancer models 150. This mechanism identifies a local osteoclast-derived signal capable of limiting the generation of antitumor T-cell responses.

Bone-metastasis-conditioned osteoclasts can also suppress immunity beyond the skeletal lesion. Across clinical cohorts and mouse models, osteoclast-derived osteopontin (OPN) entered the circulation, impaired T-cell recruitment, and reduced differentiation of TCF1^+^ precursor-like CD8^+^ T cells at extraosseous tumor sites 151. Blocking receptor activator of nuclear factor-κB ligand (RANKL), neutralizing circulating OPN, or deleting OPN in osteoclasts restored responsiveness to immune checkpoint blockade 151. These findings establish bone metastases as a source of systemic immune suppression rather than an isolated resistant compartment.

Tumor-derived lipid metabolites provide an additional connection between osteoclast remodeling and T-cell dysfunction. In nasopharyngeal carcinoma bone-metastasis models, sphingosine kinase 1 (SPHK1)-dependent production of sphingosine-1-phosphate (S1P) promoted osteoclast-precursor recruitment through sphingosine-1-phosphate receptor 3 (S1PR3) and expanded exhausted CD8^+^ T cell populations 152. Combined SPHK1 inhibition and PD-1 blockade produced greater antitumor activity than either treatment alone 152. Although demonstrated in a specific tumor context, this pathway shows how a tumor-derived metabolite can concurrently enhance osteoclast-dependent colonization and weaken adaptive immunity.

Together, these mechanisms show that osteolysis is not merely a consequence of skeletal metastasis. Matrix resorption releases TGF-β and hydroxyproline, which alter T cell polarization and reinforce tumor metabolic fitness, whereas osteoclast-derived Siglec-15 and OPN and tumor-derived S1P suppress local or systemic CD8^+^ T-cell responses. The bone niche can therefore promote immune checkpoint resistance through interconnected matrix-derived, metabolic, and osteoimmune pathways.

### Bone marrow: hematopoietic niche reprogramming and metabolic support

Distinct from the mineralized bone matrix, the bone marrow (BM) is a vascularized hematopoietic organ in which endothelial, mesenchymal, osteolineage, and adipocyte niches regulate hematopoietic stem and progenitor cell (HSPC) maintenance and differentiation. Hematologic malignancies and solid tumors can exploit this compartment through both local niche occupation and systemic reprogramming of hematopoietic output. These processes convert the BM from a homeostatic source of immune cells into a tumor-supportive compartment that generates immunosuppressive myeloid populations and provides metabolic substrates for malignant-cell survival.

### Vascular and stromal niche remodeling: metastatic seeding and host-niche conversion

Malignant cells can directly occupy and remodel niches normally required for hematopoiesis. Leukemic cells create abnormal vascular niches that alter the behavior and localization of normal hematopoietic progenitors 153, while disseminated prostate cancer cells compete with HSPCs for occupancy of the endosteal stem-cell niche 154. In breast cancer bone-metastasis models, disseminated tumor cells preferentially localized near type H vessels, a specialized vascular domain within the BM. During metastatic outgrowth, tumor-derived granulocyte colony-stimulating factor (G-CSF) induced extensive endothelial sprouting and vascular disorganization, whereas blockade of G-CSF receptor signaling reduced pathological vascular remodeling and skeletal metastatic burden 155. These findings indicate that BM colonization involves active conversion of pre-existing hematopoietic and vascular niches into microenvironments that favor malignant-cell survival and expansion.

### Tumor-driven myelopoiesis: progenitor programming and systemic immune suppression

Tumors can also reprogram BM hematopoiesis without directly infiltrating the marrow. In breast and lung tumor models, systemic tumor signals expanded osteoprogenitor populations, displaced hematopoietic stem cells, and promoted the expansion and activation of granulocyte-monocyte progenitors. Osteoprogenitor-derived matrix metalloproteinase 13 (MMP13) sustained the production of immunosuppressive myeloid cells even after removal of the primary tumor, whereas MMP13 inhibition accelerated immune recovery and restored the efficacy of immunotherapies 156.

More specific signaling and metabolic programs act directly at the progenitor level. In lung cancer models, IL-4 produced locally by BM basophils and eosinophils acted on IL-4 receptor alpha-positive early myeloid progenitors, transcriptionally programming the generation of tumor-promoting myeloid cells. Deletion of IL-4 receptor alpha in early progenitors-but not in mature myeloid cells-reduced tumor burden, and disruption of this BM signaling axis enhanced immunotherapy responses 157. Independently, lung tumors induced oxidative stress and increased chromatin accessibility for nuclear factor erythroid 2-related factor 2 (NRF2) in BM myeloid progenitors. This cytoprotective program enhanced myeloid expansion, attenuated interferon responsiveness, and persisted during differentiation into immunosuppressive monocyte-derived macrophages. Genetic or pharmacological NRF2 inhibition restored T cell and natural killer-cell activity and enhanced PD-1 blockade in preclinical models 158.

Together, these studies show that tumors actively preconfigure myeloid-cell fate in the BM, thereby ensuring the continued delivery of immunoregulatory cells to peripheral tumor sites. Bone marrow output can therefore influence immunotherapy response even when malignant cells are located outside the marrow.

### Adipocyte-derived lipids: metabolic support for marrow malignancies

Bone marrow adipocytes provide a second form of tumor support by serving as a locally accessible lipid reservoir. In acute myeloid leukemia (AML), malignant blasts induce hormone-sensitive lipase phosphorylation and lipolysis in adjacent BM adipocytes, enabling the transfer of free fatty acids to leukemia cells. Coculture also increases fatty acid-binding protein 4 (FABP4) expression in both adipocytes and AML cells. Genetic or pharmacological FABP4 inhibition reduced adipocyte-supported AML proliferation, while disruption of FABP4 or carnitine palmitoyltransferase 1A (CPT1A) improved survival in AML models 159.

Independent studies showed that BM adipocytes activate AMP-activated protein kinase (AMPK) and a fatty acid oxidation-associated transcriptional program in acute monocytic leukemia cells, reducing spontaneous apoptosis and supporting malignant-cell survival under nutrient and oxygen stress 160. Thus, adipocytes are not passive occupants of the marrow cavity but metabolically active niche cells that enable leukemia cells to acquire and oxidize exogenous fatty acids. This lipid-transfer axis primarily supports malignant-cell persistence and adaptation to metabolic stress. Its direct effects on local T cell metabolism have not yet been established.

Together, BM remodeling contributes to tumor progression through three complementary processes: occupation and conversion of hematopoietic niches, progenitor-level programming of systemic immunosuppressive myelopoiesis, and adipocyte-derived metabolic support for marrow-resident malignancies. Among these mechanisms, biased myeloid output provides the most direct connection to resistance to immune checkpoint blockade, whereas vascular and adipocyte remodeling sustain local colonization and malignant-cell survival.

Across organs, immune resistance does not arise from a single metabolite or universal nutrient deficit. Instead, tissue-specific physiology interacts with tumor-intrinsic adaptation to determine whether antitumor immune cells can enter, persist, and remain functionally competent. The brain primarily selects for metabolic autonomy and metabolite-mediated immune suppression. The liver combines physiological tolerance with bile acid- and ammonia-dependent T-cell dysfunction. Bone couples osteolysis to matrix-derived and osteoclast-mediated immune regulation. Bone marrow reprograms hematopoietic and adipocyte niches to sustain suppressive myeloid output and tumor-cell survival. These organ-dependent mechanisms shape the immune-cell populations available for reinvigoration by checkpoint blockade. The shared and organ-specific features of these immune-metabolic niches are summarized in Table 2, which distinguishes baseline physiological constraints, tumor adaptations, dominant immune bottlenecks, relevance to immune checkpoint blockade, and translational limitations across spatial contexts.

## Systemic axis: host metabolic and temporal states shaping immune checkpoint blockade responsiveness

The Systemic Axis describes how host-level metabolic and temporal states establish the baseline conditions under which antitumor immunity and ICB operate. Whereas the preceding axes describe constraints arising from local resource availability, intracellular organelle homeostasis, and tissue or organ architecture, the Systemic Axis captures how organism-wide physiology reshapes immune-cell reserve, substrate availability, systemic signaling, and the temporal opportunity for therapeutic response. Within this framework, obesity and cancer cachexia represent opposing disturbances of host energy balance, whereas gut microbial ecology and circadian organization modify the composition and timing of systemic immune-metabolic signals.

## Obesity: checkpoint-dependent immune dysfunction and parallel metabolic resistance

Obesity shapes ICB response through two partially independent programs: it can deepen checkpoint-dependent immune dysfunction that remains therapeutically reversible, while also activating metabolic and spatial resistance mechanisms that may persist despite checkpoint blockade. Chronic inflammatory and endocrine cues primarily support the first program, whereas lipid excess, adipose-derived metabolites, and adipose-to-tumor signaling can impair cytotoxic immune cells, protect malignant cells from lethal stress, and reinforce immune exclusion. The response to ICB therefore depends on the relative dominance of these programs within a specific tumor and host context.

Checkpoint-dependent immune dysfunction provides a mechanistic basis for the improved ICB outcomes observed in some obesity-associated settings. Obesity-associated leptin-signal transducer and activator of transcription 3 (STAT3) signaling increases PD-1 expression and promotes T-cell dysfunction. In experimental tumor models, this state of elevated PD-1 expression remains susceptible to PD-1 blockade 161. Obesity also induces PD-1 expression on tumor-associated macrophages (TAMs), where PD-1 signaling suppresses macrophage glycolysis, phagocytosis, antigen presentation, and T cell-stimulatory activity. PD-1 blockade can therefore restore macrophage metabolism and antitumor function in addition to reinvigorating dysfunctional T cells 162. Consistent with this checkpoint-responsive component, of obesity-associated immune dysfunction, overweight and obesity were independently associated with longer progression-free survival (PFS) and overall survival (OS) in a retrospective cohort of patients with endometrial cancer treated with ICB, with particularly strong associations in copy-number-high/TP53-abnormal tumors 163. A prospective pan-tumor cohort similarly associated obesity with improved PFS and OS, while identifying fewer circulating effector T cells, reduced cytotoxicity-marker expression, and increased exhaustion-marker expression in patients with obesity 164. Together, these findings support a checkpoint-responsive component of obesity-associated immune dysfunction in selected tumor settings.

Obesity simultaneously activates resistance mechanisms outside the PD-1 axis. In natural killer (NK) cells, peroxisome proliferator-activated receptor alpha/delta (PPARα/δ)-dependent lipid accumulation disrupts cellular metabolism and prevents the trafficking of cytotoxic granules to the immune synapse 165. At the tumor-cell level, adipocyte-derived glutathione (GSH) is transferred to breast cancer cells through scavenger receptor class B member 2 (SCARB2), activating the ADP-ribosylation factor 1-mechanistic target of rapamycin complex 1 (ARF1-mTORC1) axis, strengthening antioxidant defenses, and reducing susceptibility to T cell-mediated killing 166. In obesity-associated HCC, fatty acid-binding protein 5 (FABP5) supports adaptation to lipid excess and limits lipid peroxidation, whereas FABP5 suppression promotes ferroptosis and shifts the intratumoral immune compartment toward pro-inflammatory macrophage remodeling and increased CD8^+^ T cell activity 167. At the tissue level, extracellular vesicles (EVs) released by obese visceral adipose tissue (VAT) activate a cathepsin A-pseudouridine-mast-cell axis in PDAC, promoting fibrosis, T cell exclusion, and resistance to ICB 168. Together, these mechanisms can constrain the effectiveness of checkpoint reinvigoration by reducing immune-effector competence, lowering tumor-cell susceptibility to lethal stress, or preventing immune access to malignant cells.

Clinical heterogeneity is better resolved by tumor context and body composition than by body mass index (BMI) alone. BMI cannot distinguish visceral from subcutaneous adiposity, skeletal-muscle quantity or quality, or disease-related weight loss. In a multicenter cohort of 1,471 patients with advanced melanoma receiving first-line anti-PD-1 therapy alone or in combination with antibodies targeting CTLA-4, overweight and obesity were not associated with survival outcomes. Instead, a higher VAT index was associated with shorter OS, whereas greater skeletal-muscle density was associated with longer OS 169. These findings differ from the favorable associations reported in endometrial cancer and the smaller pan-tumor cohort 163, 164, indicating that tumor type, molecular subtype, adipose-tissue distribution, and muscle quality modify the relationship between obesity and ICB outcome.

Obesity modifies the architecture of ICB resistance rather than acting as a uniformly favorable or adverse biomarker. Checkpoint-dependent immune dysfunction may remain susceptible to therapeutic reinvigoration, whereas immune-cell lipotoxicity, tumor-cell stress adaptation, and adipose-driven immune exclusion can persist despite checkpoint blockade.

## Cancer cachexia: inter-organ catabolism and reduced checkpoint responsiveness

Cancer cachexia is a tumor-driven systemic catabolic syndrome characterized by involuntary loss of skeletal muscle, with or without adipose-tissue depletion, that cannot be fully explained by reduced food intake or reversed by conventional nutritional support 170. Tumor-derived mediators activate parallel neural and peripheral programs that suppress appetite, impair adipose-tissue regeneration, disrupt skeletal-muscle perfusion and integrity, and reprogram hepatic metabolism 171-176. Rather than constituting a single sequential cascade, these organ-specific responses converge to reduce systemic metabolic reserve and limit the capacity of the host to sustain effective antitumor immunity during treatment.

Circulating inflammatory and stress signals initiate coordinated dysfunction across multiple organs. IL-6 acts on neurons in the area postrema to alter autonomic and behavioral responses associated with cachexia 171, whereas growth differentiation factor 15 (GDF15) engages glial cell line-derived neurotrophic factor family receptor alpha-like (GFRAL) in the hindbrain to promote anorexia and systemic catabolism 172. In adipose tissue, tumor-derived macrophage migration inhibitory factor (MIF) signals through atypical chemokine receptor 3 (ACKR3), impairing adipocyte differentiation and limiting the restoration of lipid stores 173. Skeletal-muscle loss is likewise driven by more than proteolysis alone. Activin A-peroxisome proliferator-activated receptor gamma coactivator 1-alpha (PGC1α) signaling disrupts muscle endothelial function and reduces capillary density 174, while macrophage-derived tumor necrosis factor-like weak inducer of apoptosis (TWEAK) further promotes muscle atrophy in pancreatic cancer models 175. The liver also actively contributes to this inter-organ program: cachexia-associated repression of the circadian regulator REV-ERBα reprograms hepatocyte transcription and increases the production of catabolic hepatokines that promote wasting in peripheral tissues 176. Together, these pathways establish cachexia as a coordinated failure of host metabolic homeostasis rather than an isolated loss of body mass.

Hepatic failure to mount an appropriate ketogenic response can link systemic catabolism to immunotherapy resistance through glucocorticoid-mediated immune suppression. In pre-cachectic mouse models of colorectal and pancreatic cancer, tumor-derived IL-6 suppresses hepatic PPARα, limiting ketogenesis during caloric insufficiency 177. The resulting relative hypoketonemia activates a systemic glucocorticoid stress response that suppresses multiple intratumoral immune programs. Consistent with this mechanism, experimentally increasing circulating corticosterone was sufficient to abolish the response of pancreatic tumors to immunotherapy 177. These findings indicate that reduced ketogenesis impairs therapeutic tumor control primarily by activating a hepatic-glucocorticoid immunosuppressive axis, rather than simply by depriving T cells of ketone bodies as an alternative fuel.

Clinical studies support the relevance of this resistance-associated host state. In a prospective observational cohort of patients with metastatic non-small cell lung cancer (NSCLC) receiving PD-1 or PD-L1 inhibitors, baseline cancer cachexia was associated with a lower treatment response rate and independently predicted shorter PFS and OS 178. A larger multicenter study of patients with advanced NSCLC and a PD-L1 tumor proportion score of at least 50% similarly found that cachexia was associated with unfavorable outcomes in patients receiving either pembrolizumab monotherapy or an immune checkpoint inhibitor combined with chemotherapy. The addition of chemotherapy provided limited benefit in the cachectic population 179. The clinical importance of cachexia also extends beyond lung cancer. Among patients with esophageal squamous cell carcinoma treated with ICB, pretreatment cachexia was associated with shorter PFS, time to progression, and OS, while patients with persistent or irreversible cachexia experienced the poorest outcomes 180.

Cancer cachexia reduces checkpoint responsiveness by coupling the loss of systemic metabolic reserve to hepatic metabolic reprogramming and glucocorticoid-mediated suppression of intratumoral immunity. Experimental and clinical evidence therefore supports cachexia as an active resistance-associated host state rather than a passive consequence of weight loss.

## Gut microbiome: ecological and metabolic control of checkpoint sensitivity and resistance

The gut microbiome shapes ICB through community-level ecological functions and metabolite outputs rather than through a universal set of beneficial taxa. Early clinical studies identified distinct microbial configurations associated with response to PD-1-based therapy, including *Akkermansia muciniphila* and *Bifidobacterium longum* 181-183. Although the specific taxa differed across cohorts, transfer of responder-derived microbiota into germ-free or antibiotic-treated mice improved checkpoint sensitivity and antitumor immunity 181-183, providing functional evidence that the community can transmit treatment-associated phenotypes. Consistent with this ecological interpretation, metagenomic analysis organized bacterial species into co-abundance networks and generated a topological score that was validated in independent NSCLC and genitourinary cancer cohorts 184. ICB responsiveness therefore appears to reflect specific community configurations in which individual taxa act within broader functional networks.

Clinical intervention studies further indicate that resistance-associated microbial states can be modified. In 13 patients with advanced solid tumors that had progressed during anti-PD-1 therapy, responder-derived fecal microbiota transplantation (FMT) combined with continued checkpoint blockade produced disease control in six patients, including one partial response and five cases of stable disease 185. This study provides a clinical signal that microbiome remodeling can restore checkpoint sensitivity in a subset of refractory patients. A related microbiome-remodeling strategy was evaluated in the randomized phase 2a TACITO trial in treatment-naive patients with metastatic renal cell carcinoma receiving pembrolizumab plus axitinib. Although donor FMT did not significantly improve the prespecified 12-month PFS rate, the donor-FMT group had a median PFS of 24 months compared with 9 months in the placebo-FMT group and a numerically higher objective response rate 186. Associations between outcome and the acquisition or loss of specific strains, rather than overall donor engraftment, further suggest that therapeutic benefit depends on the ecological changes achieved rather than on nonspecific increases in microbial diversity.

Microbial metabolites connect these ecological states to systemic and intratumoral immune function. Short-chain fatty acids (SCFAs) illustrate the context dependence of this regulation. Butyrate can enhance the memory potential of antigen-activated CD8^+^ T cells 187, whereas SCFAs can promote both effector and regulatory T-cell differentiation through histone deacetylase (HDAC) inhibition and modulation of the mechanistic target of rapamycin-ribosomal protein S6 kinase (mTOR-S6K) pathway 188. Accordingly, elevated systemic SCFAs have been associated with reduced efficacy of CTLA-4 blockade 189, consistent with the capacity of butyrate to promote regulatory T-cell differentiation 190. Microbiota-derived inosine supports checkpoint activity through a complementary mechanism. In experimental tumors, *Bifidobacterium pseudolongum*-derived inosine enhanced therapy through adenosine A2A receptor-dependent signaling under concurrent immune activation 191, while activated CD8^+^ T cells could also use inosine as an alternative carbon source under glucose restriction 192. Microbial metabolites can therefore alter ICB response by regulating both immune-cell differentiation and metabolic adaptation.

The gut microbiome shapes ICB resistance through community ecology and metabolic function rather than through isolated beneficial or detrimental taxa. Resistance-associated communities may fail to sustain effector-cell fitness and instead reinforce regulatory immune programs, whereas targeted ecological remodeling may improve or restore checkpoint sensitivity in selected settings.

## Chrono-immunometabolism: circadian gating of immune access and checkpoint resistance

The core circadian oscillator is organized around transcriptional feedback loops involving brain and muscle ARNT-like protein 1 (BMAL1), circadian locomotor output cycles kaput (CLOCK), period (PER), and cryptochrome (CRY). These loops are reinforced by nuclear receptors such as REV-ERBα and coupled to nicotinamide adenine dinucleotide (NAD^+^) cycling and mitochondrial oxidative metabolism 193-195. In tumor models, rhythmic migration of DCs to tumor-draining lymph nodes and circadian expression of the co-stimulatory molecule CD80 generate time-dependent priming of tumor-antigen-specific CD8^+^ T cells. Synchronizing immunotherapy with the phase of maximal dendritic-cell activity consequently improved tumor control 196.

Circadian gating continues at the point of immune-cell entry into the tumor. Endothelial BMAL1 regulates the rhythmic expression of intercellular adhesion molecule 1 (ICAM-1), producing daily variation in CD8^+^ T-cell infiltration and effector function. Aligning PD-L1 blockade or chimeric antigen receptor (CAR) T cell therapy with the phase of greater T cell access improved therapeutic efficacy in mouse tumor models 197. In parallel, disruption of the intestinal epithelial clock alters inflammatory cytokine production, neutrophil recruitment, and the rhythmic accumulation of PD-L1-expressing myeloid-derived suppressor cells (MDSCs). Anti-PD-L1 treatment was most effective when synchronized with the phase of greatest PD-L1-expressing MDSC abundance 198. Circadian timing can therefore influence resistance at two complementary levels: by restricting when effector T cells can enter the tumor and by determining when suppressive myeloid populations dominate the tumor microenvironment.

Tumors can also disrupt circadian coordination beyond the local tumor microenvironment. Lung adenocarcinoma distally reprograms hepatic circadian homeostasis, including repression of REV-ERBα and disruption of rhythmic metabolic output 199. This inter-organ clock disruption suggests a systemic route through which malignant disease may weaken the temporal coordination of host metabolism and immunity. Rather than merely shifting a single treatment window, sustained circadian disruption may prolong the periods during which immune trafficking, metabolic support, and tumor immune control are poorly aligned.

Clinical studies support the relevance of this temporal regulation. In the Melanoma Outcomes Following Immunotherapy (MEMOIR) cohort, receiving at least 20% of ICB infusions after 16:30 was associated with shorter OS 200. A subsequent study-level meta-analysis of 13 studies involving 1,663 patients with advanced cancers found that earlier ICB administration was associated with longer OS and PFS 201. These findings identify treatment timing as a potentially modifiable determinant of checkpoint responsiveness. However, the optimal therapeutic window is likely to depend on tumor type, treatment regimen, and the patient's circadian phase rather than on a universal clock-time cutoff.

Chrono-immunometabolism creates recurrent windows of checkpoint sensitivity by coordinating antigen presentation, T-cell entry, and the temporal abundance of suppressive myeloid populations. Treatment outside these permissive phases, or tumor-driven disruption of host clocks, can convert transient immune misalignment into more persistent resistance.

Collectively, obesity, cancer cachexia, gut microbial ecology, and circadian organization establish the host-level metabolic and temporal context in which ICB operates. These states determine whether checkpoint-dependent dysfunction remains reversible, whether sufficient physiological reserve is available to sustain immune activity, whether microbial functions support immune-cell fitness, and when effector cells can access tumors. The Systemic Axis therefore connects organism-level physiology with the local resource, organellar, and spatial constraints described above and provides the conceptual basis for restoring immune metabolic adaptability across scales.

## Therapeutic prioritization: clinical readiness and chrono-metabolic scheduling

The preceding sections define immune-metabolic resistance as a multiscale problem involving extracellular resource constraints, organelle stress, tissue architecture, and systemic host states. Therapeutic translation therefore cannot proceed by targeting each metabolic pathway in isolation. A rational strategy must instead prioritize interventions according to clinical readiness, biological fit, safety, feasibility, and timing relative to immune activation. In this section, we organize metabolic and metabolism-adjacent interventions into a therapeutic framework that distinguishes practice-ready combinations, trial-ready systemic modifiers, specialized early clinical strategies, unsuccessful or non-confirmatory translations, and *ex vivo* engineering approaches. This structure provides the basis for a Chrono-Metabolic Scheduling Model that links each intervention to the phase in which it is most likely to modify resistance: pretreatment qualification, sequential conditioning, concurrent relief of active suppression, rescue or rechallenge, or adoptive-cell manufacturing. In this model, chrono-metabolic scheduling refers broadly to aligning metabolic intervention with the biological phase of therapy, rather than solely to circadian or time-of-day scheduling.

## Decision principles: safety qualification, biological matching, and temporal positioning

Within this therapeutic framework, metabolic intervention should be guided by four linked judgments: whether the patient can tolerate metabolic modulation, whether the intervention matches the dominant resistance mechanism, whether the supporting evidence is strong enough for clinical advancement, and when the intervention should be applied relative to immune activation. These principles shift the therapeutic discussion from a catalogue of targets to a prioritized framework for restoring immune metabolic fitness.

Safety qualification is the first step, particularly for systemic interventions that impose additional nutritional or energetic stress. Fasting-mimicking diets, ketogenic diets, and amino acid restriction may be biologically attractive, but they are not automatically low-risk interventions. Patients with cancer cachexia, sarcopenia, impaired nutrient intake, progressive weight loss, metabolic comorbidities, or limited organ reserve may lose the physiological capacity needed to sustain antitumor immunity and ongoing cancer therapy. Cancer nutrition and cachexia guidelines therefore support systematic nutritional assessment and individualized management before restrictive dietary strategies are initiated 202, 203. In this setting, safety screening should guide patient eligibility, intervention intensity, and the duration of metabolic stress.

Biological matching then determines whether the intervention addresses the relevant lesion in the relevant compartment. The same nutrient pathway or stress-response program may support malignant-cell survival, effector T cell activity, regulatory immune-cell adaptation, or host tissue homeostasis. Systemic inhibition is most rational when tumor dependence can be separated from immune and host requirements. When this separation is limited, transient exposure, local delivery, or cell-restricted manipulation may provide a more favorable therapeutic index. An intervention is therefore most appropriate when the targeted constraint is measurable, the affected compartment is reachable, and the metabolic programs required for antitumor immunity are preserved.

Evidence maturity and temporal positioning should be considered together. Randomized clinical benefit in defined indications supports practice-ready use, whereas early clinical activity, observational associations, or preclinical efficacy mainly justify prospective testing. Negative trials are equally informative because they show that pathway relevance alone does not establish therapeutic dependence. Timing should follow the biology of the resistance mechanism. Interventions that require gradual remodeling of host metabolism, vascular function, or microbial ecology are best evaluated as conditioning strategies before or early during ICB. Interventions that relieve continuously active extracellular suppression may be aligned with concurrent T cell reinvigoration. Metabolic optimization of adoptive T-cell products belongs to the *ex vivo* manufacturing phase. This framework is intended to guide therapeutic prioritization and trial design rather than prescribe a universal treatment schedule. Representative interventions are therefore organized in Table 3 according to clinical-readiness decision, dominant resistance context, evidence boundary, most defensible timing relative to immune checkpoint blockade, and principal translational limitation.

## Practice-ready niche modulation: vascular targeting and concurrent checkpoint blockade

Dysfunctional tumor vasculature forms a clinically actionable link between spatial exclusion and metabolic stress. Poorly organized vessels restrict lymphocyte entry and drug delivery, impair perfusion, and sustain hypoxia within the tumor microenvironment. These conditions reduce the likelihood that checkpoint-reinvigorated T cells can enter tumors and maintain effective antitumor function 204-207. Among the strategies discussed in this section, vascular-targeted combinations currently represent the most clinically mature example of microenvironmental modulation integrated with ICB. Although these regimens were not developed as metabolic therapies in the narrow sense, they provide the strongest clinical example of therapeutically modifying a vascular and hypoxic niche that constrains ICB efficacy.

This priority is supported by randomized clinical evidence in defined indications. In unresectable hepatocellular carcinoma, atezolizumab plus bevacizumab improved overall survival and progression-free survival compared with sorafenib in IMbrave150 208. In advanced renal cell carcinoma, pembrolizumab plus axitinib and nivolumab plus cabozantinib improved survival and disease-control outcomes compared with sunitinib in KEYNOTE-426 and CheckMate 9ER, respectively 209, 210. In advanced endometrial cancer after platinum-based therapy, lenvatinib plus pembrolizumab improved progression-free and overall survival compared with physician-choice chemotherapy in KEYNOTE-775 211. These data support vascular-targeted ICB combinations as practice-ready strategies within their validated tumor types, treatment lines, and eligibility conditions.

The relevance of these regimens to metabolic resistance is supported by mechanistic studies, but their clinical benefit should not be reduced to a single mechanism. Appropriate modulation of abnormal tumor vessels can improve perfusion, reduce hypoxia, and facilitate immune-cell access, while antitumor immune responses may reciprocally reinforce vascular normalization 204, 205. Hypoxia, especially in the context of persistent antigenic stimulation, promotes mitochondrial stress and dysfunctional T cell states 93. In preclinical models, reducing tumor-cell oxygen consumption has also decreased intratumoral hypoxia and enhanced programmed cell death protein 1 blockade 212. Together, these findings identify vascular function and oxygen availability as important determinants of whether ICB-induced immune activation can be translated into effective tumor control. However, the benefit of individual clinical regimens likely reflects combined effects on angiogenic signaling, tumor cells, stromal architecture, myeloid populations, and immune-cell entry rather than a universal vascular-normalization mechanism.

The best-supported temporal position for these regimens is concurrent administration with ICB, because overlapping exposure was built into the validated clinical combinations 208-211. A short vascular-targeting lead-in remains biologically plausible, as preclinical studies suggest that vessel normalization may occur within a limited therapeutic window 204, 205. However, the timing and durability of this window are likely to vary with drug class, dose, tumor architecture, and organ context. Direct comparisons of concurrent initiation and preconditioning schedules are therefore needed before a separate lead-in strategy can be recommended.

Clinical implementation is established but not simple. Bevacizumab targets vascular endothelial growth factor (VEGF), whereas axitinib, cabozantinib, and lenvatinib inhibit distinct and partially overlapping receptor tyrosine kinases. Their effects on perfusion, tumor cells, stroma, and immune populations are therefore not interchangeable. Excessive angiogenic inhibition may also worsen perfusion, indicating that controlled modulation of vascular signaling is more relevant than maximal pathway suppression. These regimens require monitoring for vascular, hepatic, gastrointestinal, and immune-related toxicities, and multitargeted kinase inhibitors often require dose interruption or modification. Accordingly, vascular-targeted combinations anchor the highest clinical-readiness tier, but only within the indications in which clinical benefit has been demonstrated.

## Accessible systemic modifiers: infusion timing, dietary context, and metabolic conditioning

Accessible metabolic interventions occupy a trial-ready but not practice-ready tier of immunotherapy development. This group includes low-burden variables, such as ICB infusion timing and habitual dietary context, as well as protocolized strategies that impose a defined metabolic state through fasting, ketogenesis, or nutrient restriction. Their appeal lies in feasibility: they can be incorporated into prospective studies more readily than vascular-targeted combinations, microbiome products, or engineered cell therapies. Feasibility, however, does not establish ICB sensitization. These interventions should therefore be evaluated as modifiers of the conditions in which ICB operates rather than as routine components of immunotherapy.

Low-burden modifiers are the easiest to integrate into clinical workflows, but their current evidence is mainly associative. In the retrospective MEMOIR study, a greater proportion of late-day ICB infusions was associated with poorer overall survival in advanced melanoma 200, and a subsequent study-level meta-analysis similarly associated earlier infusion times with more favorable outcomes across advanced cancers 201. These findings make infusion timing a clinically testable scheduling variable, but not an established chronotherapeutic standard. Habitual diet provides a parallel example. In patients with melanoma receiving ICB, higher dietary fiber intake was associated with longer progression-free survival, particularly among patients reporting sufficient fiber intake without probiotic supplementation, and mouse experiments supported a functional link between dietary fiber, microbial ecology, and antitumor immunity 213. Together, these data justify prospective validation of treatment timing and dietary context, while leaving open whether clock time, patient-specific circadian phase, microbial metabolites, or broader nutritional patterns drive the observed associations.

Protocolized systemic interventions produce stronger metabolic perturbations and therefore require stricter safety and adherence controls. A cyclic five-day fasting-mimicking diet was feasible in nutritionally selected patients with cancer, reduced circulating glucose and growth-factor concentrations, altered peripheral immune populations, and was associated with changes in intratumoral immune-cell composition in a translational breast-cancer cohort 214. These findings show that fasting-mimicking diets can induce systemic and immunological effects in selected patients, but they do not establish improved ICB outcomes. Ketogenic interventions provide a related rationale through ketone availability and T cell metabolic adaptation. Ketogenesis-derived β-hydroxybutyrate supports programs associated with CD8^+^ memory T cell development 215, and ketogenic diets or β-hydroxybutyrate supplementation enhanced or restored responses to PD-1 blockade in preclinical tumor models 216. Clinical testing should therefore determine whether these approaches are best used as pre-ICB conditioning or concurrent metabolic modulation, and whether either schedule provides clinical benefit, with objective verification of metabolic exposure.

Amino acid restriction most clearly illustrates why systemic metabolic intervention must be temporally controlled. Dietary methionine restriction can alter one-carbon metabolism in humans and has shown antitumor activity in mouse and patient-derived tumor models 217. In immunocompetent models, intermittent methionine deprivation increased tumor-cell susceptibility to ferroptosis and CD8^+^ T cell killing and enhanced checkpoint blockade through ChaC glutathione-specific gamma-glutamylcyclotransferase 1 (CHAC1)-mediated glutathione degradation 218. However, nutrient restriction can also create compensatory immune escape: a serine- and glycine-free diet increased cytotoxic T-cell accumulation but induced PD-L1 lactylation 219. These findings support short, reversible conditioning windows as a testable strategy, while cautioning against continuous deprivation without evidence that effector immunity and host reserve are preserved.

Overall, accessible interventions should be advanced through structured scheduling trials rather than adopted simply because they are easy to implement. Infusion timing and dietary fiber require prospective validation of observational signals, whereas fasting-mimicking, ketogenic, and amino-acid-restricted diets require protocolized testing with prespecified nutritional, metabolic, and immune monitoring. Their immediate value is to test whether systemic metabolic conditions can be shifted before or during ICB in a way that improves antitumor immunity without compromising patient reserve.

## Microbiome and metabolite-directed strategies: specialized delivery and biomarker-dependent targeting

Specialized microbiome-directed and metabolite-directed interventions have entered different stages of early clinical translation. FMT, standardized live bacterial products, and adenosine A2A receptor (A2AR) blockade have generated patient-level signals in combination with ICB, whereas monocarboxylate transporter 1 (MCT1) inhibition has so far established human targetability without demonstrated ICB sensitization. Compared with dietary or scheduling interventions, these strategies require defined products, pharmacological target engagement, or biomarker-informed patient selection. Their current translational value lies in identifying trial-prioritized approaches for patients in whom microbial ecology or metabolite-mediated suppression materially contributes to ICB resistance.

Microbiome conditioning provides the clearest patient-level example of a sequential strategy. Phase I studies in anti-PD-1-refractory melanoma showed that responder-derived FMT followed by anti-PD-1 reinduction was associated with renewed clinical activity in a subset of patients 220, 221. This concept was later extended to FMT before anti-PD-1 therapy in advanced melanoma 222, and the phase II FMT-LUMINate trial reported objective responses after healthy-donor FMT followed by anti-PD-1-based therapy in non-small cell lung cancer and melanoma 223. Randomized evidence remains non-confirmatory: in TACITO, donor FMT combined with pembrolizumab and axitinib did not significantly improve the prespecified 12-month progression-free survival endpoint, although secondary efficacy measures favored donor FMT 186. Because FMT remains biologically and operationally variable, it should be developed as a trial-prioritized conditioning strategy before ICB initiation or ICB rechallenge rather than as routine microbiome therapy.

Standardized live bacterial products may reduce some of the variability of donor-derived FMT. In two small randomized phase I studies in treatment-naive metastatic renal cell carcinoma, daily oral CBM588 was added to either nivolumab plus ipilimumab or cabozantinib plus nivolumab 224, 225. Both studies reported preliminary clinical signals favoring CBM588, although microbiome endpoints were not consistently met and the sample sizes were insufficient to establish efficacy. Because CBM588 is administered continuously with ICB-based therapy, it is best viewed as a concurrent microbiome-modulating strategy whose clinical value remains to be defined in larger efficacy and pharmacodynamic studies.

Pharmacological targeting of adenosine signaling offers a more spatially defined approach to metabolite-mediated immune suppression. In treatment-refractory renal cell carcinoma, the oral A2AR antagonist ciforadenant showed that adenosine signaling could be inhibited safely in patients and produced early antitumor activity alone and with PD-L1 blockade 226. Because adenosine-mediated suppression can persist during T cell reinvigoration, A2AR blockade provides a mechanistic rationale for concurrent development with ICB, particularly in biomarker-defined tumors in which adenosine signaling actively sustains resistance.

Lactate transport targeting is less mature in the ICB setting. First-in-human testing of the MCT1 inhibitor AZD3965 established the feasibility of pharmacologically targeting lactate transport in patients with advanced cancer 227. However, this trial did not test ICB sensitization. MCT1 inhibition should therefore be described as a clinically targetable metabolic pathway without established ICB-combination efficacy.

Collectively, these strategies define an intermediate but heterogeneous stage of translation between accessible systemic modifiers and fully experimental metabolic engineering. Their development should now move from broad clinical expansion toward standardized delivery, pharmacodynamic confirmation in the relevant compartment, biomarker-defined enrollment, and schedule-specific testing.

## Negative metabolic translation: pathway relevance, target engagement, and patient selection

Negative and non-confirmatory trials are essential for defining the boundaries of metabolic intervention. They show that a pathway may contribute to resistance to ICB without becoming a clinically exploitable vulnerability in every tumor type, dose, schedule, or patient population. Their value is therefore not only to exclude ineffective regimens, but also to clarify what future metabolic-ICB trials must prove: that the targeted pathway is active in the relevant compartment, that the intervention modifies it sufficiently, and that this modification improves antitumor immunity rather than merely altering a circulating biomarker.

IDO1 inhibition provides the clearest example. Tryptophan depletion and kynurenine accumulation can suppress effector T cell activity, providing a strong rationale for combining IDO1 inhibition with PD-1 blockade. However, in the randomized phase III ECHO-301/KEYNOTE-252 trial, epacadostat plus pembrolizumab did not improve progression-free or overall survival compared with pembrolizumab alone in unselected patients with unresectable or metastatic melanoma 228. This failure does not invalidate tryptophan catabolism as a resistance mechanism, but it shows that pathway relevance is not equivalent to therapeutic dependence. Effective translation requires patient selection, adequate intratumoral target modulation, and confirmation that the targeted metabolic pathway is a dominant driver of resistance in the treated population.

Metformin illustrates a different limitation: a biologically plausible lead-in does not necessarily create an effective conditioning phase. Metformin can reduce tumor-cell oxygen consumption and has been proposed to relieve hypoxia-associated T-cell dysfunction, providing a rationale for use before or during ICB. In treatment-refractory microsatellite-stable (MSS) metastatic colorectal cancer, however, a phase II study administered metformin for 14 days before adding nivolumab and observed no objective responses, leading to early termination for futility 229. Although this design explicitly tested a metabolic lead-in before checkpoint blockade, the result indicates that temporal separation alone is insufficient. A conditioning phase is meaningful only if it produces a measurable change in the resistance mechanism that limits ICB efficacy.

Together, these examples establish a practical standard for future metabolic-ICB combinations. Trials should enrich for patients with the candidate metabolic lesion, include pharmacodynamic sampling in the relevant tumor or immune compartment, and link the proposed schedule to a measurable biological transition. When timing is the central hypothesis, lead-in and concurrent schedules should be compared directly where feasible. Metabolic scheduling becomes therapeutically meaningful only when the intervention demonstrably alters the resistant state and improves clinical outcome.

## Therapeutic T-cell engineering: *ex vivo* metabolic resilience and product fitness

The preceding strategies aim to modify the patient environment in which antitumor immunity operates. Metabolic optimization of adoptive T-cell products addresses the same resistance problem from a different direction: instead of correcting every hostile feature of the tumor niche *in vivo*, therapeutic T cells can be prepared *ex vivo* to better tolerate nutrient limitation, hypoxia, suppressive metabolites, oxidative stress, and chronic antigenic stimulation after infusion. This strategy does not represent a conventional metabolic intervention scheduled before or during ICB. Rather, it shifts the timing and site of intervention to the manufacturing phase, where T cell state can be modified before the product encounters the tumor microenvironment.

The most clinically advanced evidence comes from manufacturing-stage pathway modulation. bb21217 is a B-cell maturation antigen (BCMA)-directed CAR T-cell product manufactured in the presence of the phosphoinositide 3-kinase (PI3K) inhibitor bb007 to enrich memory-like T cells. In the phase I CRB-402 study of patients with relapsed or refractory multiple myeloma, bb21217 showed clinical activity, and early-memory features of the infused product were associated with greater expansion and deeper responses 230. Although this evidence comes from hematologic malignancy rather than solid tumors, it demonstrates that metabolic or signaling modulation during manufacturing can be incorporated into a patient-tested cellular product and linked to therapeutic performance.

Reversible manufacturing interventions may offer a practical route to improve T-cell fitness without adding a permanent genetic component. Incorporating protein kinase B (AKT) inhibition into a clinical-grade AUTO1 manufacturing process enriched stem-like and central-memory populations, increased polyfunctionality, and improved antitumor activity in preclinical models 231. Direct metabolic control has also been explored through inhibition of the mitochondrial pyruvate carrier (MPC). Exposure to the MPC inhibitor MITO-66 during manufacturing generated stem cell-like memory CD19 CAR-T cells from healthy donors and patients with B-cell malignancies and improved tumor control in mouse models 232. These approaches place metabolic optimization within the *ex vivo* manufacturing window, where reproducibility, product yield, potency, and compatibility with existing release criteria become central translational requirements.

Stable genetic engineering may be more appropriate when the dominant metabolic pressure is expected to persist after infusion. Forkhead box O1 (FOXO1) overexpression promoted a stem-like CAR-T cell state, improved mitochondrial fitness and persistence, and enhanced antitumor activity in models using cells from healthy donors and patients with solid tumors 233. A complementary strategy is to engineer T cells to metabolically detoxify a defined suppressive pathway. Kynureninase (KYNU)-modified CAR-T cells degraded immunosuppressive kynurenine, maintained stronger proliferative and cytotoxic activity under kynurenine-rich conditions, and improved tumor control in IDO1-expressing tumor models 234. These studies illustrate two distinct engineering principles: reinforcing intrinsic T cell metabolic fitness and reducing exposure to a suppressive metabolite within the local tumor environment.

The translational readiness of these approaches depends on the depth of engineering required. Temporary culture modulation is closer to implementation because it changes product phenotype without permanently altering the genome. Stable metabolic engineering offers greater mechanistic specificity but introduces additional requirements for control of transgene expression, genomic safety, long-term stability, and interaction with CAR signaling. Across both tiers, improved metabolic fitness must be evaluated together with expansion, persistence, effector differentiation, cytokine production, exhaustion risk, and safety. Engineering therapeutic T cells for metabolic resilience therefore extends the therapeutic framework from environmental correction to cellular adaptation: when metabolic resistance cannot be fully removed from the tumor niche, the therapeutic cell product may be designed to function within it.

## Chrono-metabolic scheduling: pretreatment conditioning, concurrent relief, and rechallenge design

The therapeutic value of a metabolic intervention depends not only on the pathway it targets, but also on when the intervention is applied relative to immune activation. We therefore propose a Chrono-Metabolic Scheduling Model that organizes metabolic strategies into five practical positions: pretreatment qualification and lesion mapping, sequential conditioning before ICB, concurrent relief of active suppression during ICB, rescue or rechallenge after resistance, and *ex vivo* optimization of adoptive T-cell products. This model is not intended to prescribe a universal treatment calendar. Instead, it provides a trial-design framework in which timing is matched to the kinetics of the resistance mechanism, the target compartment, feasibility constraints, and the maturity of the supporting evidence.

Pretreatment qualification is the entry point for subsequent scheduling decisions. Before a metabolic intervention is added to immunotherapy, trials should determine whether the patient can tolerate the intervention and whether a measurable resistance constraint is present. Nutritional reserve, organ function, comorbidities, concomitant therapy, microbial context, circadian or treatment-time variables, and tumor-specific metabolic features may therefore inform eligibility and stratification. This step is particularly important for restrictive dietary interventions, systemic metabolic inhibition, and microbiome-directed strategies, which may fail or cause harm if applied without adequate physiological reserve or without a relevant targetable constraint.

Sequential conditioning is most defensible when the resistant state requires time to be remodeled before ICB can act effectively. Microbial ecology, systemic nutrient and growth-factor signaling, ketone exposure, tumor nutrient dependence, and T-cell differentiation state may shape the baseline conditions under which checkpoint blockade begins. Interventions such as fecal microbiota transplantation, fasting-mimicking diets, ketogenic strategies, or short reversible amino acid restriction should therefore be tested as conditioning approaches only when they produce measurable changes in the intended host, microbial, tumor, or immune compartment. Without such pharmacodynamic evidence, a lead-in phase remains a schedule rather than a mechanism-based intervention.

Concurrent intervention is most defensible when the suppressive pathway remains active during T cell reinvigoration, tumor entry, or effector function. Vascular dysfunction, hypoxia, extracellular adenosine signaling, acidity, and lactate-linked suppression can continue to restrain immune activity after checkpoint blockade has been initiated. Validated vascular-targeted combinations provide the strongest clinical example of this logic, whereas adenosine A2A receptor blockade, live bacterial products, and lactate-transport targeting remain at earlier stages of development. In this setting, the central trial question is whether the added intervention relieves an active barrier during ICB rather than merely changing a related systemic biomarker.

The model also accommodates combined sequential-concurrent designs. For example, a trial could test whether a short systemic conditioning phase first shifts the host or tumor metabolic state, followed by ICB with a concurrent intervention directed against persistent extracellular suppression. Such a design would directly address the question of sequential versus simultaneous metabolic intervention. It would also require two pharmacodynamic checkpoints: evidence that the conditioning phase remodeled the intended compartment before ICB, and evidence that the concurrent phase relieved an active suppressive signal during T-cell activation or tumor infiltration.

Rescue or rechallenge strategies require a different evidentiary standard. After primary resistance, acquired resistance, or relapse after an initial response, the goal is not simply to repeat a conditioning intervention later, but to identify the resistant state that has emerged and determine whether it can be reversed before ICB is continued or reintroduced. Serial sampling of tumor tissue, blood, microbiota, or metabolic markers is therefore essential. Rechallenge should be considered biologically meaningful only when the intervention demonstrably modifies the mechanism limiting renewed immune responsiveness.

*Ex vivo* optimization applies the same scheduling principle to adoptive cell therapy by shifting the intervention from the patient to the therapeutic cell product. When metabolic resistance cannot be safely corrected *in vivo*, therapeutic T cells may instead be manufactured or engineered to better tolerate nutrient limitation, hypoxia, suppressive metabolites, oxidative stress, or chronic antigenic stimulation. This strategy should be evaluated through product-level evidence, including phenotype, potency, expansion, persistence, cytokine profile, exhaustion risk, and safety.

Thus, the Chrono-Metabolic Scheduling Model directly addresses whether metabolic interventions should be administered before, during, after, or outside the *in vivo* ICB treatment sequence. Within this framework, sequential treatment may be prioritized for prospective testing when baseline remodeling is required, whereas concurrent treatment may be more appropriate when suppression persists during immune activation. Combined sequential-concurrent designs may be considered when both baseline remodeling and active suppression relief are mechanistically required; rescue or rechallenge may be evaluated when an acquired resistant state can be defined and reversed; and manufacturing-stage optimization may be considered when cellular adaptation is more feasible than environmental correction. Together with the evidence and feasibility categories summarized in Table 3, this model reframes metabolic intervention as a set of biologically timed and evidence-calibrated strategies for prospective testing rather than as uniform adjuncts to immunotherapy.

## Conclusion

The central message of this review is that resistance to ICB cannot be understood solely as insufficient relief of inhibitory receptor signaling. Durable antitumor immunity requires a permissive immune-metabolic architecture in which tumor-reactive T cells can obtain substrates, maintain organelle fitness, enter and persist within tumor tissue, withstand suppressive metabolites and oxidative stress, and operate within a host state capable of supporting immune recovery. When one or more of these conditions is compromised, checkpoint-released or partially reinvigorated T cells may remain unable to convert receptor blockade into sustained tumor control.

The multiscale framework developed here explains why metabolic resistance is difficult to treat through simple pathway-based interventions. Local nutrient competition, enzymatic depletion, lipid stress, suppressive metabolites, and ionic stress can restrain TCR signaling, biosynthesis, redox homeostasis, and functional persistence. Mitochondrial and endoplasmic reticulum dysfunction can further limit the recoverability of exhausted T cells, whereas intercellular mitochondrial transfer can redistribute metabolic fitness or damage between immune and malignant cells. Stromal, vascular, and organ-specific niches determine whether metabolically competent lymphocytes can reach malignant cells and remain functional, while obesity, cachexia, microbial ecology, and circadian organization shape the systemic baseline on which ICB acts. These levels should not be viewed as independent modules or as a strictly linear cascade. Rather, they form an interdependent constraint network in which failure at one scale can narrow the therapeutic opportunity available at another.

This framework also emphasizes that metabolic pathways are rarely uniformly suppressive or uniformly therapeutic. The same nutrient, metabolite, or stress-response pathway may support tumor-cell survival, sustain effector T-cell function, enable regulatory immune-cell adaptation, or preserve host tissue homeostasis, depending on cellular compartment, disease context, and timing. This principle is evident across amino acid restriction, lipid handling, lactate biology, obesity-associated immunity, microbiome-derived metabolites, and potassium-dependent immune suppression or *ex vivo* conditioning. The translational question is therefore not simply whether a pathway is metabolic or whether it participates in resistance. The more relevant question is whether it represents a dominant, measurable, and safely targetable bottleneck in a defined patient population.

This context dependence has direct therapeutic implications. Metabolic interventions should not be developed as generic adjuncts to ICB. They should be prioritized according to clinical evidence, patient metabolic reserve, target compartment, feasibility, and timing relative to immune activation. Practice-ready vascular-targeted combinations differ fundamentally from trial-prioritized dietary or microbiome-directed strategies, early metabolite-targeted approaches, and preclinical metabolic engineering of adoptive T-cell products. Similarly, pretreatment conditioning, concurrent blockade of active suppression, rescue or rechallenge after resistance, and *ex vivo* manufacturing modification address different biological problems. The Chrono-Metabolic Scheduling Model proposed in this review is therefore best understood as an evidence-anchored trial-design framework rather than a fixed treatment calendar.

Future studies should move beyond associating metabolic features with ICB response and should test whether specific metabolic lesions causally limit immunotherapy in the relevant compartment. This will require patient stratification by dominant resistance mechanism, paired pharmacodynamic sampling, spatial metabolomics, single-cell multi-omics, functional immune assays, and prospective comparisons of sequential and concurrent schedules when timing is central to the hypothesis. Safety should be embedded into this biological design, particularly for restrictive dietary interventions, systemic metabolic inhibitors, and patients with cachexia, sarcopenia, metabolic comorbidities, impaired nutrient intake, or limited organ reserve.

Taken together, the available evidence supports a shift in how tumor immunometabolism is interpreted. Metabolism is not merely a background barrier that weakens immunotherapy, nor is it simply a collection of new targets to combine with checkpoint inhibitors. It defines the conditions under which immune activation can become durable immune control. The next stage of metabolism-informed immunotherapy will depend on identifying which metabolic constraint is dominant, where it operates, whether it can be safely modified, and when intervention is most likely to widen the window for effective antitumor immunity.

## Figures and Tables

**Figure 1:**
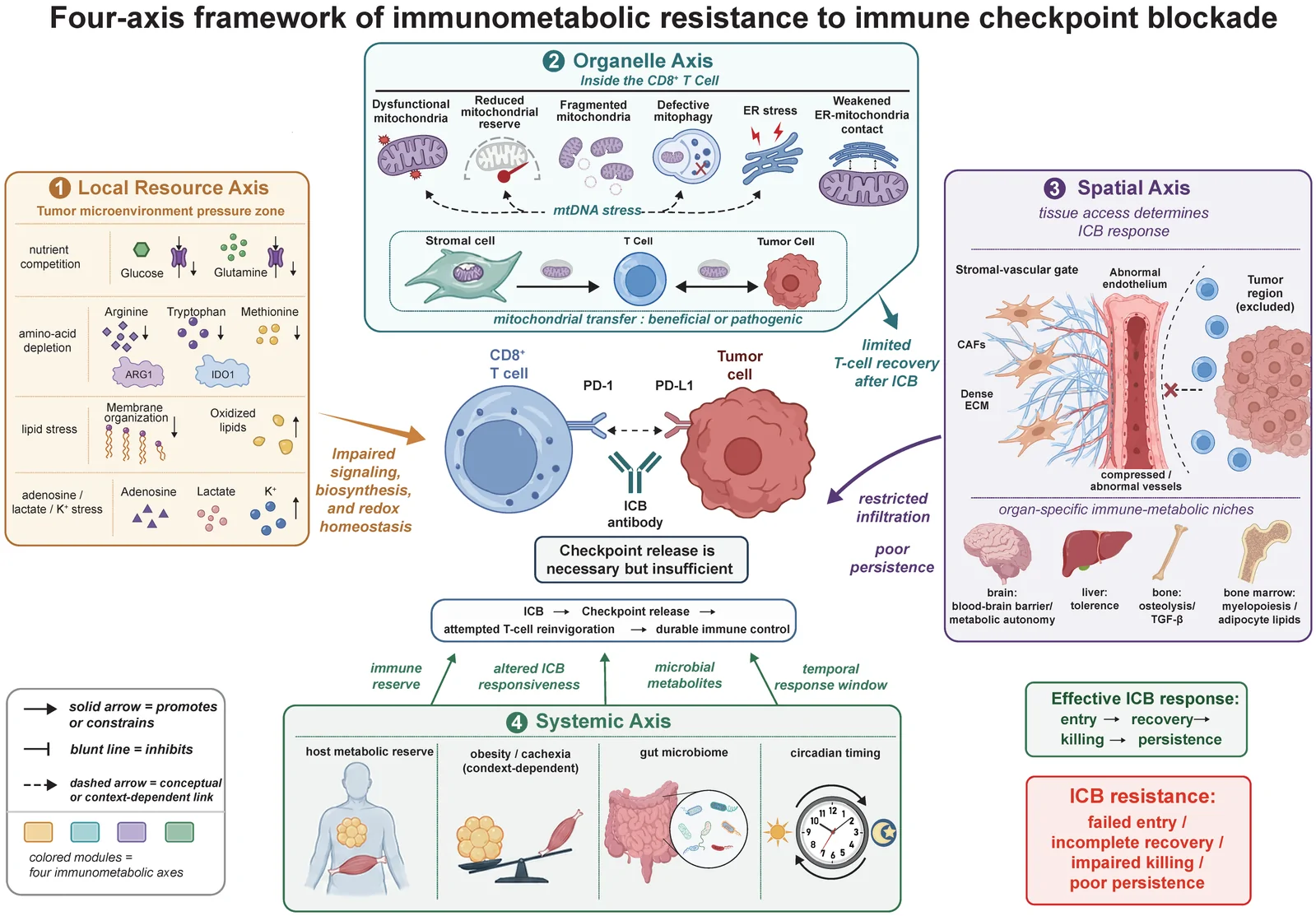
Four-axis framework of immunometabolic resistance to immune checkpoint blockade. ICB releases inhibitory receptor signaling but does not by itself ensure durable antitumor immunity. This framework organizes immunometabolic barriers to ICB responsiveness into four interconnected axes. The Local Resource Axis comprises nutrient competition, amino-acid depletion, lipid stress, suppressive metabolites, and ionic constraints. The Organelle Axis comprises impaired mitochondrial reserve, defective mitophagy, mitochondrial DNA stress, endoplasmic reticulum (ER) stress, ER-mitochondria dysfunction, and context-dependent mitochondrial transfer. The Spatial Axis comprises stromal-vascular exclusion and organ-specific immune-metabolic niches. The Systemic Axis comprises host metabolic reserve, obesity, cachexia, gut microbial ecology, and circadian organization. The mitochondrial DNA (mtDNA) stress component depicts a plausible, context-dependent connection among mitochondrial dysfunction, cytosolic DNA sensing, senescence-associated signaling, and ICB resistance, rather than a single continuous pathway established in one tumor model. Together, these constraints limit T-cell entry, functional recovery, cytotoxicity, and persistence after checkpoint release, thereby promoting incomplete immune reinvigoration and ICB resistance. These functions are interdependent requirements for an effective ICB response rather than obligatory steps in a fixed temporal sequence.

**Figure 2:**
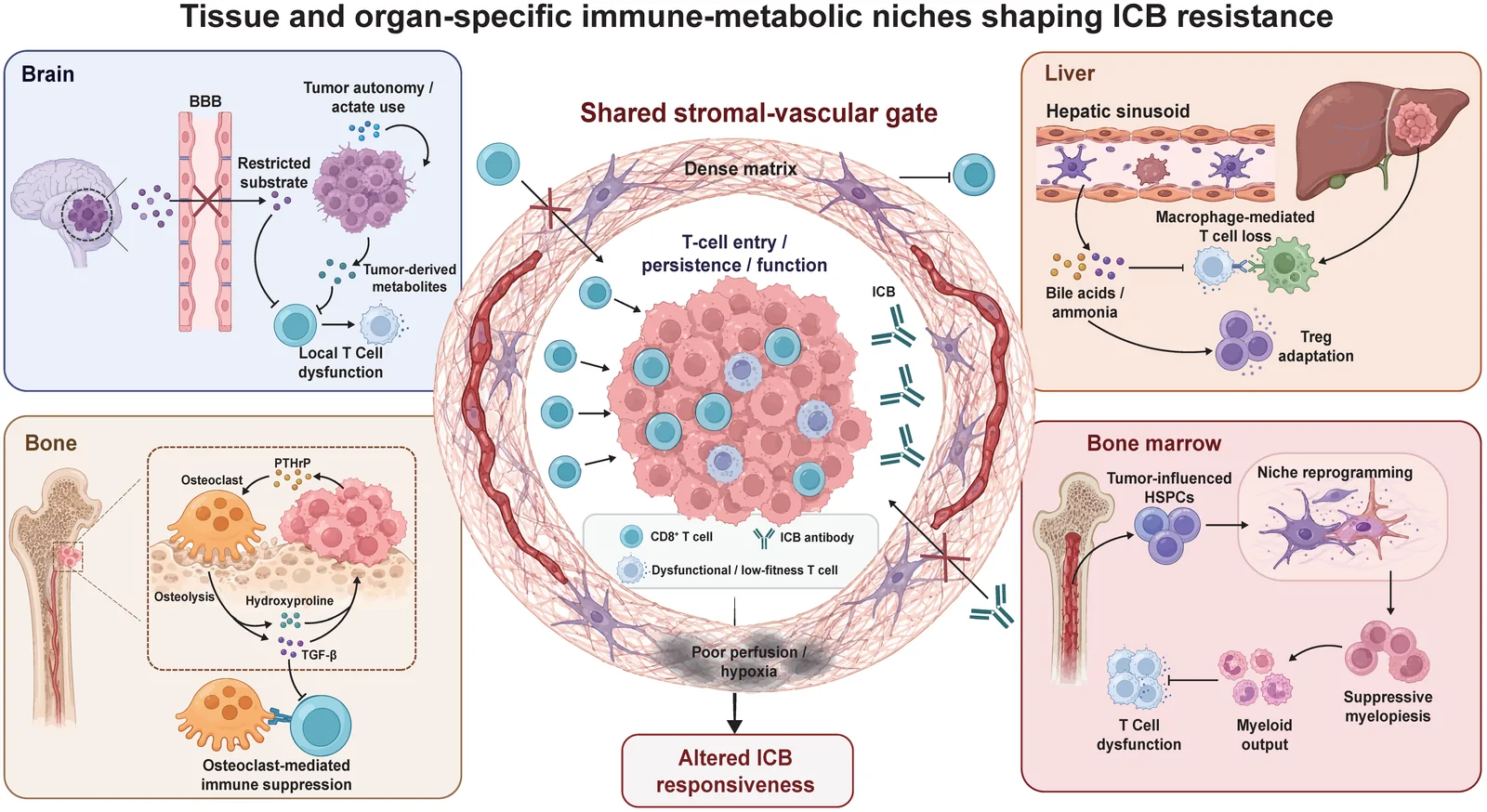
Tissue and organ-specific immune-metabolic niches shaping immune checkpoint blockade resistance. The Spatial Axis illustrates how tissue architecture and organ physiology constrain ICB by regulating T-cell access, persistence, and function. A shared stromal-vascular gate, shaped by CAFs, dense extracellular matrix, vessel compression, abnormal endothelium, and impaired perfusion, limits therapeutic penetration and CD8^+^ T-cell extravasation. Organ-specific niches impose additional immune-metabolic bottlenecks. In the brain, blood-brain barrier (BBB)-restricted substrates combine with tumor metabolic autonomy and glioma-derived suppressive metabolites. In the liver, tolerogenic metabolism combines with bile acid and ammonia stress, macrophage-mediated T-cell loss, and Treg adaptation. In bone, osteolysis-derived TGF-β and hydroxyproline combine with osteoclast-centered suppression. In bone marrow, niche reprogramming promotes suppressive myeloid output. Together, these niches show that spatial resistance to ICB reflects tissue-specific barriers rather than a universal metabolic defect.

**Table 1:** Multiscale immunometabolic barriers underlying resistance to immune checkpoint blockade

Axis	Resistance module	Main compartment	Bridge to ICB failure	Evidence boundary	Translational implication
Local Resource Axis	Nutrient partitioning and substrate competition	Malignant cells, myeloid cells, effector T cells	Substrate competition limits TCR signaling, biosynthesis, cytokine production, and clonal expansion after checkpoint release	Supported in selected tumor models, although nutrient competition is context-dependent rather than universal	Define the dominant limiting substrate before testing nutrient-directed interventions
	Enzymatic amino acid depletion, methionine-cycle dysregulation, and metabolite signaling	Myeloid cells, tumor cells, dendritic cells, T cells	Arginine, tryptophan, and methionine lesions impair TCR signaling, nutrient sensing, methylation, or regulatory skewing	Mechanistic chains are supported by complementary systems, but not always within the same *in vivo* ICB model	Amino acid interventions require lesion-specific design because simple supplementation or restriction may be misleading
	Lipid stress, redox failure, and ferroptotic vulnerability	CD8⁺ T cells, tumor cells, lipid-rich niches	Lipid accumulation, oxidized-lipid uptake, antioxidant failure, or cholesterol imbalance weakens persistence, membrane signaling, and ferroptosis resistance	Strong preclinical evidence supports selected lipid-redox mechanisms, although clinical translation remains context-dependent	Distinguish adaptive fatty-acid use from lipotoxic or ferroptotic stress
	Suppressive metabolites and ionic stress	T cells, regulatory T cells, myeloid cells, endothelial or stromal niches	Adenosine, lactate-linked chromatin regulation, and extracellular potassium restrain activation, transcriptional state, or effector function	Evidence ranges from receptor signaling to cell-state-specific epigenetic and ionic models	Separate receptor signaling, intracellular uptake, epigenetic encoding, and ionic conditioning rather than treating all metabolites as equivalent targets
Organelle Axis	Mitochondrial reserve and metabolic recoverability	Exhausted or chronically stimulated CD8⁺ tumor-infiltrating lymphocytes	Reduced mitochondrial biogenesis, oxidative phosphorylation, and fitness limit proliferation and cytotoxic recovery after PD-1 blockade	Evidence is mostly mechanistic and preclinical, and clinical proof that mitochondrial restoration improves ICB remains limited	Prioritize mitochondrial restoration when T-cell recoverability is the limiting lesion
	Defective quality control and senescence-associated dysfunction	CD8⁺ tumor-infiltrating lymphocytes	Failed mitophagy, mitochondrial stress, and possible mtDNA-associated senescence stabilize terminal dysfunction	Mitophagy-exhaustion links are supported, although the full mtDNA-cGAS-STING-senescence-ICB chain remains partly inferential	Frame as a recoverability barrier, not a universally established clinical target
	Direction-dependent mitochondrial transfer and organelle redistribution	T cells, tumor cells, stromal cells, immune-cell networks	Mitochondrial transfer can enhance T-cell fitness or redistribute functional and pathogenic mitochondria toward tumor advantage	Direction- and cargo-dependent effects are emerging, although therapeutic control remains at an early stage	Distinguish beneficial mitochondrial delivery from tumor-mediated mitochondrial acquisition or damage transfer
	ER-mitochondrial coupling and stress-response signaling	CD8⁺ T cells, dendritic cells, selected tumor compartments	Disrupted ER-mitochondrial coordination and maladaptive stress signaling weaken T-cell activation and dendritic-cell support	Evidence is mostly preclinical and cell-type-specific, so branch-specific stress responses should not be generalized	Targeting should be compartment-specific because the same pathway may affect T cells and antigen-presenting cells differently
Spatial Axis	Stromal and vascular barriers to immune access	Cancer-associated fibroblasts, tumor endothelial cells, extracellular matrix, perivascular niches	Stromal support, vessel compression, endothelial dysfunction, and immune-excluding vessels restrict T-cell entry and persistence	Strong mechanistic and translational rationale, with clinical validation for selected vascular-targeted combinations	Spatial remodeling is clinically actionable when poor infiltration or perfusion dominates
	Organ-specific immune-metabolic niches	Brain, liver, bone, bone marrow, resident stromal and immune populations	Tissue physiology, barriers, resident immune programs, and organ-derived metabolites shape site-specific checkpoint responsiveness	Evidence is organ- and model-specific, ranging from patient associations to preclinical models	Treat organ site as a biological variable in ICB trial design and biomarker interpretation
Systemic Axis	Host metabolic reserve and inflammatory baseline	Whole host, adipose tissue, skeletal muscle, liver, circulating immune compartment	Obesity, cachexia, sarcopenia, and catabolism reshape immune reserve, checkpoint dependence, tolerance, and recovery capacity	Clinical associations are stronger than direct causal intervention evidence in many settings	Use nutritional and metabolic assessment as a safety and stratification gate before systemic metabolic intervention
	Microbial and temporal regulation of immune responsiveness	Gut microbiome, microbial metabolites, circadian immune programs, systemic signaling networks	Microbial ecology and circadian timing regulate immune availability, metabolite exposure, lymphocyte trafficking, and response windows	Human associations and early interventional signals exist, but causality and standardization remain incomplete	Microbiome- and timing-based strategies should remain evidence-calibrated and prospectively tested

Abbreviations: cyclic GMP-AMP synthase (cGAS), endoplasmic reticulum (ER), immune checkpoint blockade (ICB), mitochondrial DNA (mtDNA), programmed cell death protein 1 (PD-1), stimulator of interferon genes (STING), and T-cell receptor (TCR).

**Table 2:** Spatial and organ-specific immune-metabolic niches shaping antitumor immunity and checkpoint responsiveness

Niche	Baseline constraint	Tumor adaptation	Immune bottleneck	ICB relevance	Translational implication
Stromal-vascular interface as a shared spatial gate	Matrix density, perfusion gradients, endothelial metabolism, and vascular immune-gatekeeping	Fibroblast-derived substrates, desmoplastic vessel compression, and immune-excluding or lymphocyte-recruiting endothelium	Restricted perfusion, impaired drug delivery, limited T-cell extravasation, and effector-cell exclusion or loss	ICB may fail when reinvigorated T cells cannot enter, distribute within, or persist in tumors	Spatial remodeling is actionable in selected settings, but benefit should not be reduced to vascular normalization alone
Brain	Blood-brain barrier transport, neural nutrient demand, and restricted extracellular substrates	Biosynthetic autonomy, acetate use, and glioma-derived metabolites that reprogram macrophages or suppress cytotoxic lymphocytes	Local T-cell access, proliferation, and effector function may remain constrained despite tumor metabolic autonomy	ICB efficacy may depend on local access, antigen presentation, and metabolic support within brain lesions	Brain-specific vulnerabilities require lesion- and cell-type-specific targeting because systemic metabolic assumptions may not apply
Primary liver tumor niche	Portal antigen exposure, immune tolerance, bile acid metabolism, nitrogen disposal, and steatohepatitis-associated inflammation	Inflammatory T-cell dysfunction, bile acid stress, ammonia-rich niches, and regulatory T-cell metabolic adaptation	Tumor-reactive T cells may be dysfunctional, metabolically stressed, or outcompeted by metabolically adapted regulatory T cells	In steatohepatitis-associated hepatocellular carcinoma, PD-1 expression may mark tissue-damaging or nonproductively activated T cells	Etiology, bile acid composition, ammonia burden, and metabolic liver disease should inform ICB-response interpretation
Liver metastasis and systemic hepatic immune suppression	Hepatic filtering of activated lymphocytes and macrophage-rich tolerogenic niches	Macrophage programs that delete activated T cells or sustain exhaustion in fibrotic regions	Systemic loss or dysfunction of activated T cells impairs extrahepatic antitumor immunity	Liver metastases can suppress ICB responses beyond the hepatic lesion	Liver involvement should be treated as a systemic immunological variable, not only an anatomic metastatic site
Bone	Mineralized collagen matrix stores TGF-β and undergoes osteoclast-mediated remodeling	Osteolysis releases matrix-derived TGF-β and hydroxyproline, while osteoclast-centered programs reinforce tumor growth and suppression	Altered T-cell polarization, osteoclast-mediated suppression, and matrix-derived metabolic support	Bone lesions may show reduced checkpoint responsiveness when osteolysis-driven immune remodeling dominates	Strategies may need to combine ICB with osteolysis-, TGF-β-, or osteoclast-directed modulation. Clinical validation remains limited
Bone marrow hematopoietic niche	Endothelial, mesenchymal, osteolineage, and hematopoietic stem and progenitor cell niches regulate immune-cell production	Tumors remodel vascular or stromal niches and bias progenitor output toward suppressive myeloid programs	Sustained suppressive myelopoiesis reduces the immune-cell pool available for productive ICB response	Bone marrow reprogramming can affect ICB responsiveness even when dominant lesions are outside marrow	Hematopoietic niche status may become a stratification variable, but therapeutic reversal remains investigational
Bone marrow adipocyte-rich metabolic niche	Marrow adipocytes provide lipid reservoirs under nutrient and oxygen stress	Marrow-resident malignancies induce lipolysis and capture fatty acids for oxidative metabolism and survival	Malignant-cell persistence is metabolically supported. Direct effects on local T cells are less established	This niche may promote tumor-cell survival, but direct contribution to checkpoint resistance remains insufficiently established	Frame as tumor-supportive metabolic adaptation unless stronger evidence links it directly to ICB failure

Abbreviations: immune checkpoint blockade (ICB), programmed cell death protein 1 (PD-1), and transforming growth factor beta (TGF-β)

**Table 3:** Clinical readiness, evidence boundaries, and chrono-metabolic timing of immunometabolic strategies for immune checkpoint blockade

Strategy	Readiness	Resistance context	Evidence boundary	Timing	Main limitation	Refs.
Pretreatment nutritional and cachexia screening	Practice-enabling safety step	Low metabolic reserve, cachexia, sarcopenia, impaired intake, or limited organ reserve	Guideline-supported assessment plus clinical associations with ICB outcomes	T0: qualification before restrictive or systemic metabolic intervention	Does not directly reverse resistance but defines eligibility, risk, intervention intensity, and monitoring	180, 202, 203
Vascular-targeted combinations with ICB	Practice-ready in validated indications	Vascular dysfunction, impaired perfusion, hypoxia, angiogenic signaling, and spatial immune exclusion	Level 1 evidence in defined regimens and indications	T2: concurrent with ICB. Lead-in remains investigational	Use is regimen- and indication-specific. Toxicity management is required. Benefit cannot be reduced to vascular normalization	204-211
ICB infusion timing and circadian alignment	Trial-ready but not practice-ready	Circadian variation in antigen presentation, trafficking, endothelial adhesion, and tumor infiltration	Level 4 with preclinical support	T2: scheduling variable during ICB	Retrospective confounding remains a concern. Clock time may not reflect patient-specific circadian phase	193, 196-198, 200, 201
Dietary fiber and microbial dietary context	Trial-ready but not practice-ready	Microbiome-associated ICB variation and microbial metabolite availability	Level 4/5: patient association plus preclinical support	T0-T2: baseline assessment and treatment-period management	Diet-microbiome causality is difficult to isolate. The intervention is not established as an ICB-sensitizing prescription	181-183, 187, 189, 191, 213
Fasting-mimicking diet	Trial-ready but not practice-ready	Systemic nutrient sensing, growth-factor signaling, and immune-metabolic reserve	Level 3: feasibility and immune-metabolic modulation. ICB benefit remains untested	T1: monitored conditioning before or early during ICB	Requires nutritional selection, adherence monitoring, and recovery between cycles. ICB efficacy remains unconfirmed	202, 203, 214
FMT-based microbiome conditioning	Trial-prioritized early clinical strategy	Microbiome-associated resistance or failed anti-PD-1 response	Level 2/3 mixed: phase I/II signals with randomized phase II non-confirmatory evidence	T1: pretreatment conditioning. T3: rescue or rechallenge	Donor selection, screening, standardization, engraftment variability, and combination safety remain barriers	185, 186, 220-223
Standardized live bacterial supplementation, such as CBM588	Trial-prioritized early clinical strategy	Microbiome modulation during ICB-based therapy	Level 3: small randomized phase I studies	T2: concurrent oral administration with ICB-based regimens	Evidence is product-specific. Microbiome endpoints are inconsistent, and efficacy studies are underpowered	224, 225
Adenosine A2A receptor blockade	Trial-prioritized early clinical strategy	Extracellular adenosine-mediated suppression during T-cell reinvigoration	Level 3: phase I activity and targetable-pathway evidence	T2: concurrent relief of active suppression	Requires biomarker-defined adenosine-dependent tumors. Confirmatory efficacy remains limited	226
Ketogenic diet or β-hydroxybutyrate supplementation	Investigational / preclinical	Glucose-restricted contexts and ketone-supported CD8⁺ T-cell memory programs	Level 5: mechanistic and preclinical ICB-combination evidence	T1-T2: conditioning or concurrent modulation	Requires standardized diet and ketosis verification. Tumor ketone use, weight change, and adherence remain unresolved	215, 216
Intermittent amino acid restriction	Investigational / preclinical	Tumor nutrient dependence, redox vulnerability, and ferroptotic sensitization	Level 5 for ICB synergy. Human metabolic modulation has been shown for methionine restriction	T1: short, reversible conditioning	May impair immune proliferation or host reserve. Optimal duration and patient selection remain undefined	217-219
MCT1 inhibition or lactate-transport targeting	Investigational	Lactate transport, acidic stress, and lactate-dependent immune adaptation	Level 3 for human targetability. No established ICB-combination efficacy	T2: concurrent hypothesis with ICB	MCT4 compensation, immune/normal-tissue transport, and lack of ICB-sensitization data limit interpretation	227
IDO1 inhibition with epacadostat	Not supported as tested	Tryptophan catabolism and kynurenine-mediated suppression	Level 1 negative: randomized phase III evidence against the tested regimen	T2 tested and not supported in unselected melanoma	Pathway relevance did not translate into clinical dependence. Target engagement and patient selection remain unresolved	228
Metformin lead-in before nivolumab	Not supported as tested	Tumor oxygen consumption and hypoxia-associated T-cell dysfunction	Level 3 negative: phase II evidence against the tested schedule and population	T1 lead-in followed by T2 combination tested and not supported	Lead-in rationale did not produce objective responses in refractory microsatellite-stable colorectal cancer	207, 212, 229
Manufacturing-stage T-cell modulation	Specialized *ex vivo* translational strategy	Suboptimal differentiation, limited persistence, and metabolic fragility of adoptive cell products	Level 3 for patient-tested CAR T-cell manufacturing and Level 5 for preclinical optimization	Tx: *ex vivo* manufacturing	Product yield, potency, comparability, release criteria, and manufacturing reproducibility must be revalidated	230-232
Stable metabolic engineering of therapeutic T cells	Investigational / preclinical *ex vivo* strategy	Persistent suppressive metabolites or intrinsic T-cell metabolic dysfunction after infusion	Level 5: preclinical proof of principle	Tx: *ex vivo* genetic engineering	Requires transgene control, genomic safety, long-term stability, CAR-signaling compatibility, cost control, and scalability	233, 234

Evidence levels are defined as follows. Level 1 denotes randomized phase III evidence or confirmatory clinical evidence, including either demonstrated clinical benefit or definitive evidence against a tested regimen. Level 2 denotes randomized phase II evidence or a prospective phase II efficacy signal. Level 3 denotes phase I evidence, early clinical proof of concept, patient-tested feasibility, or clinical targetability. Level 4 denotes an observational clinical association. Level 5 denotes preclinical proof of principle. “Negative” indicates evidence against the tested drug, schedule, population, or regimen, not necessarily against the biological pathway. Timing definitions are as follows. T0 denotes pretreatment qualification and lesion mapping. T1 denotes conditioning before immune checkpoint blockade initiation or during the earliest treatment window. T2 denotes concurrent intervention during immune checkpoint blockade. T3 denotes maintenance, rescue, or rechallenge after resistance. Tx denotes *ex vivo* manufacturing or engineering of adoptive T-cell products. Abbreviations: adenosine A2A receptor (A2AR), chimeric antigen receptor T cell (CAR T cell), fecal microbiota transplantation (FMT), immune checkpoint blockade (ICB), indoleamine 2,3-dioxygenase 1 (IDO1), monocarboxylate transporter 1 (MCT1), monocarboxylate transporter 4 (MCT4), and programmed cell death protein 1 (PD-1).

## Data Availability

No datasets were generated or analyzed during the current study.

## Abbreviations

AA: arachidonic acid
ACAT1: acyl-CoA:cholesterol acyltransferase 1 (SOAT1)
ACSS2: acetyl-CoA synthetase 2
ADO: adenosine
ARG1: arginase 1
ASCT2: alanine-serine-cysteine transporter 2
ASS1: argininosuccinate synthase 1
BAs: bile acids
BBB: blood-brain barrier
BM: bone marrow
BMAs: bone marrow adipocytes
BMSCs: bone marrow stromal cells
CAAs: cancer-associated adipocytes
CAFs: cancer-associated fibroblasts
CD247: CD3ζ chain
CTCs: circulating tumor cells
DAMP: damage-associated molecular pattern
DCs: dendritic cells
DREs: dioxin response elements
ER: endoplasmic reticulum
ETC: electron transport chain
EVs: extracellular vesicles
FAK: focal adhesion kinase
FAO: fatty acid oxidation
FASN: fatty acid synthase
FFAs: free fatty acids
FMD: fasting-mimicking diet
GBM: glioblastoma
GLS2: glutaminase 2
GSH: glutathione
HA: hyaluronan
HCC: hepatocellular carcinoma
HEVs: high endothelial venules
HSPCs: hematopoietic stem and progenitor cells
ICAM-1: intercellular adhesion molecule 1
ICB: immune checkpoint blockade
ICC: intrahepatic cholangiocarcinoma
IFP: interstitial fluid pressure
IL-7/IL-15: interleukin-7/interleukin-15
KD: ketogenic diet
LSECs: liver sinusoidal endothelial cells
MDSCs: myeloid-derived suppressor cells
MERCs: mitochondria-endoplasmic reticulum contact sites
MUFAs: monounsaturated fatty acids
NETs: neutrophil extracellular traps
NR: nicotinamide riboside
OTC: ornithine transcarbamylase
OXPHOS: oxidative phosphorylation
P5C: pyrroline-5-carboxylate
PDAC: pancreatic ductal adenocarcinoma
PE-AA: phosphatidylethanolamine-arachidonic acid
PGE2: prostaglandin E2
PKA: protein kinase A
PRODH: proline dehydrogenase
PUFAs: polyunsaturated fatty acids
ROS: reactive oxygen species
SAM: S-adenosylmethionine
SASP: senescence-associated secretory phenotype
SCD1: stearoyl-CoA desaturase-1
SCFAs: short-chain fatty acids
SFAs: saturated fatty acids
SLC16A1: solute carrier family 16 member 1/MCT1
SLC16A3: solute carrier family 16 member 3/MCT4
SLC2A1: solute carrier family 2 member 1/GLUT1
SLC7A11: solute carrier family 7 member 11/xCT
SOAT1: sterol O-acyltransferase 1
SQSTM1: sequestosome 1/p62
TA-HEVs: tumor-associated high endothelial venules
TAMs: tumor-associated macrophages
TECs: tumor endothelial cells
TME: tumor microenvironment
TNTs: tunneling nanotubes
UPR: unfolded protein response
VAT: visceral adipose tissue
VAT-EVs: visceral adipose tissue-derived extracellular vesicles
VCAM-1: vascular cell adhesion molecule 1
